# Does miRNA Expression in the Spent Media Change During Early Embryo Development?

**DOI:** 10.3389/fvets.2021.658968

**Published:** 2021-04-08

**Authors:** Paul Del Rio, Pavneesh Madan

**Affiliations:** Biomedical Sciences, Ontario Veterinary College, University of Guelph, Guelph, ON, Canada

**Keywords:** miRNA, culture media, IVF, early embryo health, bovine

## Abstract

Distinct miRNA populations have been detected in the spent media of *in-vitro* culture systems. However, profiling has been limited to media conditioned with blastocyst-stage embryos. Therefore, the aim of the study was to profile extracellular miRNAs throughout the pre-implantation period in bovine embryos. To achieve this, cumulus oocyte complexes were aspirated from ovaries, *in-vitro* matured, fertilized, and cultured under standard laboratory procedures to the 2-cell, 8-cell, or blastocyst stage of development. At each developmental stage, 25 μl of spent *in-vitro* culture media was collected, pooled to 300 μl, and processed for total RNA extraction. *In-vitro* culture media, which never came in contact with any embryos, were additionally processed for total RNA extraction to serve as a negative control. Following hybridization on a GeneChip miRNA 4.0 array, comparative analysis was conducted between spent media and control samples. In total, 111 miRNAs were detected in the spent media samples, with 56 miRNAs identified in blastocyst spent media, 14 miRNAs shared between 8-cell and blastocyst spent media, 7 miRNAs shared between all 3 conditions, and 6 miRNAs exclusive to 2-cell spent media. miRNA-mRNA target prediction analysis revealed that the majority of genes predicted to be regulated by the miRNAs identified in the study have roles in cellular process, metabolic process, and biological regulation. Overall, the study suggest that miRNAs can be detected in the spent media of *in-vitro* culture system throughout the pre-implantation period and the detected miRNAs may influence genes impacting early embryo development.

## Introduction

Declining cattle fertility is a widely recognized problem resulting in economic losses and culling of cattle (1). Although a multitude of factors contribute to declines in reproductive fitness, early embryonic mortality remains a major cause of infertility (2). It is estimated that 75–80% of fetal losses following successful AI occurs within this period and is more frequently observed in repeat service cattle (3). With declining successes with traditional AI, the industry has looked toward the use of IVF systems, as an alternative means of attaining embryos for transfer. According to the International Embryo Transfer Society (IETS), the period of 2013–2017 has seen consistent increases in embryos being produced, with 2017 seeing almost one million embryos produced *in-vitro* (4–8). Although IVF systems allow for the selection of potentially genetically superior gametes and embryos, its uses within the industry have been limited. With pregnancy and calving rates remaining similar to natural breeding or AI, combined with costly materials and skilled labor requirements, the mainstream use of IVF systems in the industry is yet to be seen (9). A limitation in the IVF system that needs to be addressed for its mainstream application in the industry is the methodologies used for assessing embryo viability. Currently, the industry standard is morphological assessment, which involves the embryologist to score embryos on parameters based on appearance such as cleavage rate, blastomere size, blastomere symmetry, number of blastomeres, homology of cytoplasm, and zona pellucida thickness, as outlined by the IETS (10). Although studies have shown that morphological assessment do correlate with better implantation and calving rates, the subjective nature of scoring by the embryologist makes the process inconsistent (11). Studies have shown that morphologically high-quality embryos may possess aneuploidies that can alter an embryo's overall developmental potential (1). Efforts have been made toward developing other non-invasive forms of assessment such as the characterization of miRNAs in spent media (SM) conditioned with embryos. miRNAs are small non-coding RNAs (18–22 nt) involved in post-transcriptional regulation of gene expression causing either the translational repression or controlled degradation of the mRNA (12). Binding in the 3′untranslated region is not completely complementary, therefore allowing miRNAs to have multiple gene targets across an organism (12). These gene targets have been associated to have influences in all biological systems, such as apoptosis, growth, proliferation, reproduction, and development, with expression occurring in a spatially and temporally specific manner (13).

In embryos, multiple studies have been conducted showing that miRNAs play a significant role in coordinating gene expression throughout this crucial developmental period. It has been determined that certain miRNAs are required at key stages of the pre-implantation developmental period, defined as from day 0 to day 8, with miR-25 and miR-181 being identified as being expressed in the early and late stages, respectively (14). It is also noted that a host of miRNAs may play a role in regulating genes that facilitate the processes involved in maternal to embryonic transition, often seen as one of the greatest developmental milestone in early embryonic development (14).

In light of new research, it has also been implicated that these temporal changes in miRNA expression may also be reflected in the SM. Multiple studies have shown that miRNAs can be released into the extracellular environment via containment in exosomes, apoptotic bodies, or bound to proteins. In embryos, studies conducted by Kropp et al. (15), Rosenbluth et al. (16), and Kropp et al. (17), showed that miRNAs can be detected in the SM of bovine and human IVF systems (15–17). These studies showed that there are significant differences in the miRNAs detected in the SM of degenerate embryos and embryos that successfully formed into blastocyst. This suggest that miRNAs in the SM may be used as biomarkers of early embryonic health as changes in intra-cellular miRNA expression may be reflected in the extra-cellular milieu, and that these changes may reflect the overall health or quality of the embryo.

To the best of our knowledge, profiling of miRNAs in the SM have been limited to either a few candidate miRNAs, or only examined the blastocyst stage of development. Preliminary studies conducted in our lab using previously cited candidate miRNAs suggest that miRNAs can be detected in the SM of cleavage-staged embryos. Therefore, the objective of this study is to globally profile the miRNAs present in the SM throughout the pre-implantation developmental period using a microarray approach. This method will allow for the discovery of miRNAs detected at earlier stages of embryo development, as well as discovery of miRNAs that are shared between or throughout the pre-implantation developmental period.

## Materials and Methods

### Chemicals

All chemicals were attained from Sigma-Aldrich, Oakville, ON, Canada, unless stated otherwise.

### Oocyte Collection and *in-vitro* Production of Bovine Embryos

Bovine ovaries were collected from a local abattoir (Cargill Canada, Guelph, Ontario) and transported to the laboratory in a thermo flask under phosphate buffered saline (NaCl, 136.9 mM; Na2HPO4, 8.1 mM; KCL, 1.47 mM; KH2PO4, 1.19 mM; MgCl2.6H2O, 0.49 mM) at a temperature of 35–36°C. Follicles ranging from 4 to 8 mm were aspirated using an 18G vacutainer needle and was suspended in HEPES-buffered Hams F-10 supplemented with 2% donor calf serum (PAA Laboratories Inc., ON, Canada). Cumulus oocytes complexes (COCs) were washed twice with 3 mL synthetic *in-vitro* maturation (S-IVM) media (TCM-199 plus 2% donor calf serum, Sigma-Aldrich) and washed once with 3 mL S-IVM supplemented with 0.5 g/ml of follicle stimulating hormone, 1 g/ml of luteinizing hormone and 1 g/ml of estradiol. Approximately, groups of 15–20 COCs with homogenous cytoplasm and 4–5 layers of granulosa cells were matured in 80 μl drops of S-IVM under a layer of silicone oil for 22–24 h at 38.5°C in an atmosphere of 5% CO_2_ with 100% humidity. After maturation, the COCs were washed twice with 3 ml HEPES buffered Tyrode's albumin-lactate-pyruvate medium (HEPES/Sperm TALP) supplemented with 15% BSA (0.0084 mg/ml final; fatty acid free) and washed twice with 3 mL IVF-TALP (IVF-TALP consisting of Tyrode's solution, supplemented with 15% BSA and 2 mg/ml heparin). Approximately 20 COCs were placed in 80 μl drops of IVF-TALP under a layer of silicone oil. Frozen thawed bovine sperm was prepared using swim-up technique. Thawed sperm was placed in HEPES/Sperm TALP and incubated for 45 min at 38.5°C in an atmosphere of 5% CO_2_ with 100% humidity prior to centrifugation at 200 g for 7 min. The COCs and sperm were co-incubated at a final concentration of 1.0 × 10^6^ at 38.5°C in 5% CO_2_ with maximum humidity. At 18 h post fertilization (hpf), the presumptive zygotes were denuded by gentle vortexing for 90 s, followed by washing twice with 3 ml HEPES/Sperm TALP, and once with *in-vitro* culture (IVC) media [CaCl_2_·2H_2_O, 1.17 mM; KCL, 7.16 mM; KH_2_PO_4_, 1.19 mM; MgCl_2_·6H_2_O, 0.49 mM; NaCl, 107.7 mM; NaHCO_3_, 25.07 mM, Na lactate (60% syrup), 3.3 mM; ChemiconMillipore, Billerica, MA, USA] supplemented with 50 μL of 100 × non-essential amino acids (glycine, _L_-alanine, _L_-asparagine, _L_-aspartic acid, _L_-glutamic acid, _L_-proline, _L_-serine; all 0.2 mM final), 100 μL 50x essential amino acids (_L_-arginine hydrochloride, 0.6 mM final; _L_-cysteine, 0.1 mM final; _L_-histidine hydrochlorideH_2_O, 0.2 mM final; _L_-isoleucine, 0.4 mM final; _L_-leucine, 0.4 mM final; _L_-lysine hydrochloride, 0.4 mM final; _L_-methionine, 0.1 mM final; _L_-phenylalanine, 0.2 mM final; _L_-threonine, 0.4 mM final; _L_-tyrosine, 0.2 mM final; _L_-tryptophan, 0.05 mM final; _L_-valine, 0.4 mM final), 25 μL of sodium pyruvate (0.00886 mg/ml final), 2.5 μL of gentamicin (25 mg/ml final; all from Invitrogen, Burlington, ON, Canada), and 280 μl of 15% bovine serum albumin (0.0084 mg/ml final). Approximately 30 presumptive zygotes (PZ) with homogenous cytoplasm were cultured in 30 μl of IVC media under silicone oil at 38.5°C in an atmosphere of 5% CO_2_, 5% O_2_, 90% N_2_ and cohorts of embryos were cultured to the 2-cell, 8-cell, and/or blastocyst stage of development and SM was collected at specific HPF after each desired time stage was reached (Table 1).

**Table 1:** Spent media collection schedule at various stages [adapted from Van Soom et al. (Van 18), Perkel and Madan (18)].

**Cell stage**	**Hour-Post fertilization (HPF)**
2-cell	18–30
8-cell	60–80
Blastocyst	168–192

### Collection of Spent *in-vitro* Culture Media

Prior to collection of SM at each developmental stage, developmental rates for 2-cell, 8-cell, and blastocyst formation was recorded to ensure that culture conditions were representative of average IVP production rates as reported by Van Soom et al. (19). Following this, the embryos were removed from the IVC drops, washed 5 times in PBS, and flash frozen in liquid nitrogen and stored at −80°C for future downstream gene analysis. Approximately 25 μL of SM was collected from each drop, and samples were pooled together in 1.5 mL Eppendorf tubes for each of the developmental stage analyzed. The pooled samples were flash frozen in liquid nitrogen and stored at −80°C until RNA processing. In total, 3,000 μL, 3,300 μL, and 1,250 μL of SM was collected from 2-cell, 8-cell, and blastocyst staged embryos, respectively. In addition, 1,050 μL of unconditioned media, which did not contact any embryos, was collected to serve as a negative control.

### miRNA Extraction

miRNA extraction was isolated from SM and plain media using a RNeasy mini kit (Qiagen, Hilden, Germany), as downstream array analysis required total RNA sample input. Briefly, 350 μL of SM and plain media was aliquoted to a 2.5 mL Eppendorf tube and equal volumes of QIAzol lysis reagent was added, vortexed for 20 s, and placed on the benchtop at room temperature for 10 min. This was followed by the addition of 350 μL of chloroform and incubated for 2 min at room temperature, prior to centrifugation at 12 g (15,000 RPM) at 4°C for 15 min. After, the supernatant was placed into the RNeasy min elute spin column for total RNA separation. Once all the supernatant was processed, washing steps using buffer RWT, buffer RPE, and 80% ethanol, was performed as per manufacturer protocol. The RNA was eluted using 30 μL of RNAse-free water and immediately stored in −80°C prior to microarray analysis. In total, 3 biological replicates of pooled SM from each timed stage of development and plain media was processed and prepared for microarray analysis.

### miRNA Microarray Hybridization

Microarray processing was all conducted by our colleagues at Genome Quebec (McGill University, Montreal, Quebec). Briefly, microarray profiling was conducted using the Affymetrix GeneChip miRNA 4.0 array (Affymetrix, Santa Clara, CA, USA), according to manufacturer's instructions and as described previously by Reza et al. (20). Briefly, each sample of RNA was labeled using the FlashTag Biotin RNA Labeling Kit (Genisphere, Hatfield, PA, USA), quantified, fractionated, and hybridized to the miRNA microarray. The protocol is as follows: labeled RNA is heated to 99°C for 5 min, then heated at 45°C for 5 min, prior to hybridization via constant agitation at 60 rpm for 16 h at 48°C on an Affymetrix 450 Fluidics Station. The microarray chip is washed and stained with Genechip Fluidics Station 450, prior to being scanned with the use of an Affymetrix GCS 3,000 scanner and computed using the Affymetrix Genechip command console software.

### Statistical Analysis

For Genechip microarray analysis, CEL files were imported in the Affymetrix Transcriptome Analysis Console® 4.0.2.15 (TAC) software in RMA+DMG (all organisms) mode. Comparative analysis was carried out between SM samples (2-cell, 8-cell, and blastocyst) and control (plain media) using fold-change and independent *T*-test, in which the null hypothesis was that no difference exist between the 2 groups. Probes were differentially expressed at a fold-change of ≤−2 or ≥2 (*p* < 0.05), where probe-sets were considered expressed if ≥50% of samples have a detectable above background (DABG) values below DABG threshold of <0.05 and a false discovery rate (FDR) of <0.05. All statistical test and visualization of differentially expressed genes were done using TAC software (https://www.thermofisher.com/ca/en/home/global/forms/life-science/download-tac-software.html).

#### Target Pathway Prediction of Differentially Expressed miRNAs

Functional analysis of differentially expressed miRNAs (DEM) detected at 3 SM conditions was performed using TargetScan Human 7.2 (http://www.targetscan.org/vert_71/) under Cow annotation, to construct a gene-list from the DEM. Genes with a cumulative context score of < -0.75 was included in the list. From the gene-list, gene-set enrichment analysis (GSEA) was conducted using PANTHER (http://pantherdb.org/) with Bos Taurus selected for the organism option and functional classification, under gene-ontology: Biological Processes, was conducted. Pathways with a *p* < 0.05 was considered significantly enriched.

## Results

### Differentially Expressed miRNAs in 2-Cell, 8-Cell, and Blastocyst SM

**Figure 1:**
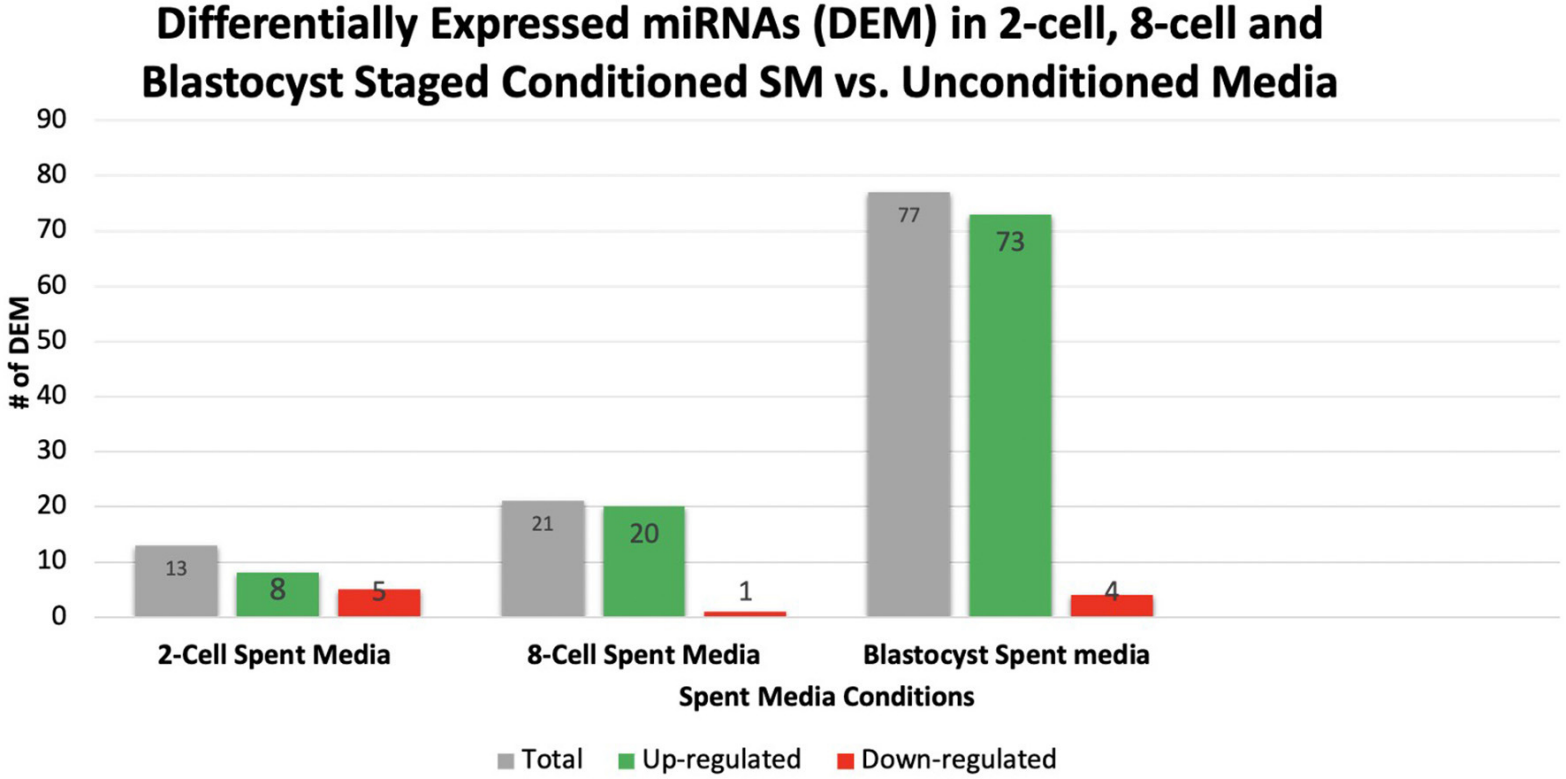
A total of 111 miRNAs were differentially expressed in 2-cell, 8-cell, and blastocyst spent media. Expression of miRNAs in spent media increased throughout the 3 conditions examined, with 13, 21, and 77 miRNAs being detected in 2-cell, 8-cell, and blastocyst spent media, respectively. The majority of miRNAs detected in spent media were blastocyst derived.

**Figure 2:**
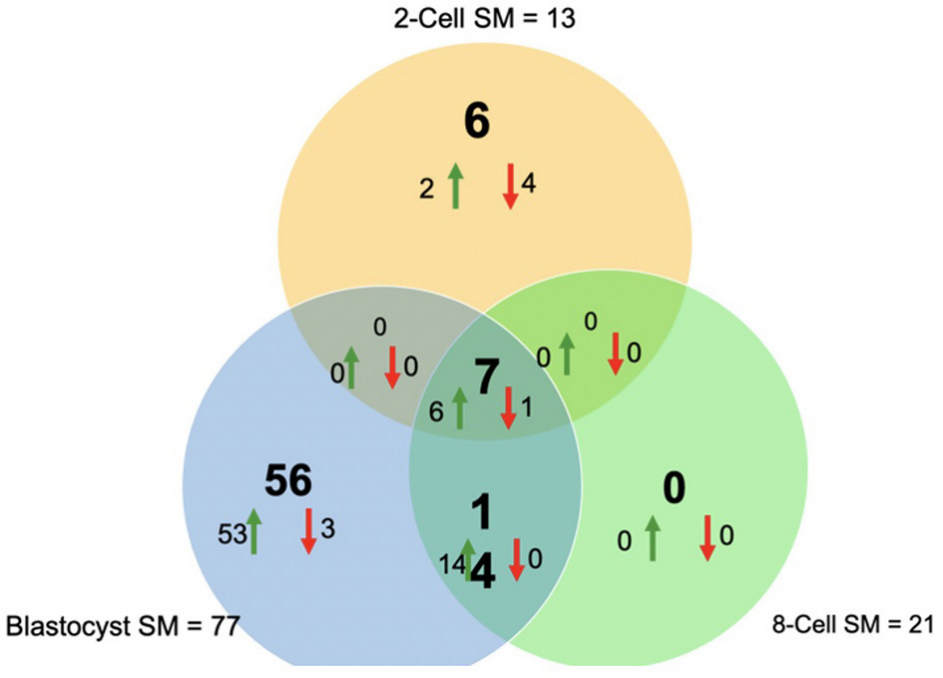
Venn diagram of differentially expressed miRNAs from 2-cell (yellow), 8-cell (green), and blastocyst SM (blue). Overlapping results showed differentially expressed miRNAs shared between 2 or more groups (14 miRNAs between 8-cell and blastocyst spent media; 7 miRNAs between all 3 spent media conditions).

**Figure 3:**
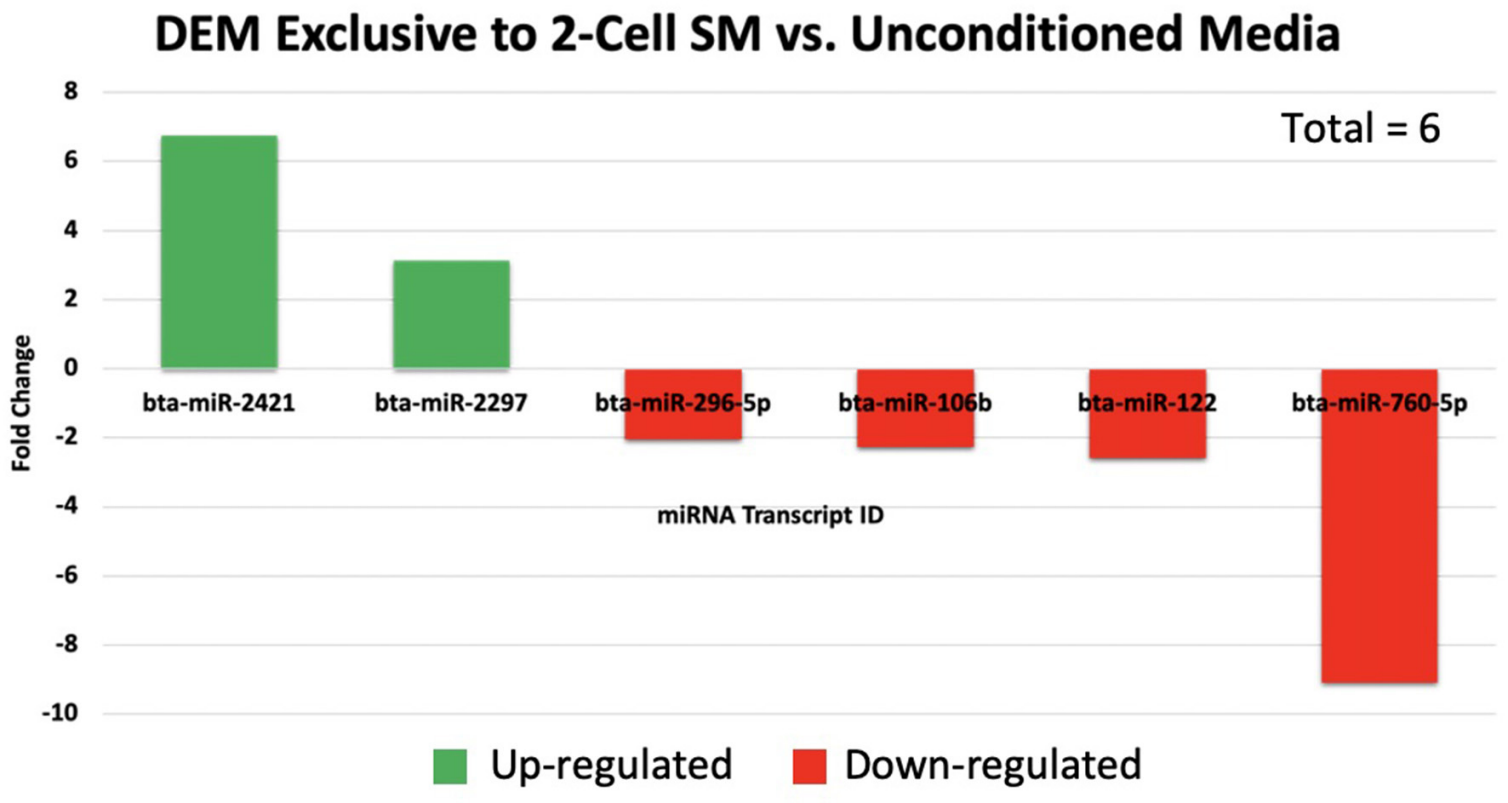
A total of 6 miRNAs were significantly expressed in 2-cell spent media. Two miRNAs were up-regulated, while 4 miRNAs were down-regulated in 2-cell spent media.

**Figure 4:**
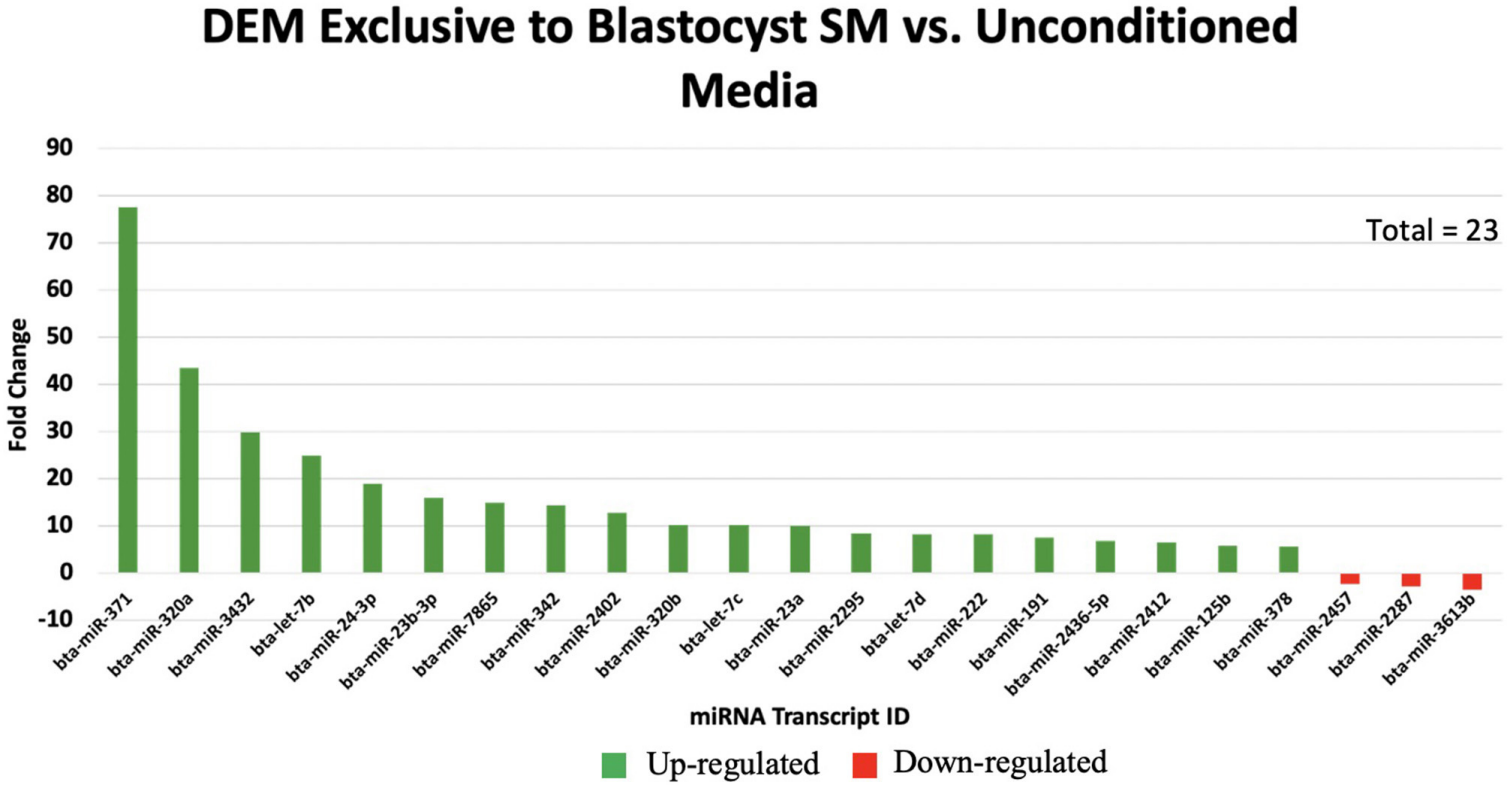
The top 20 of 53 significantly up-regulated miRNAs are graphed with the 3 significantly down-regulated miRNAs exclusive to blastocyst spent media.

Overall, a total of 111 miRNAs were differentially expressed in the SM conditioned with 2-cell, 8-cell, and/or blastocyst embryos, when compared to plain media. Thirteen miRNAs were detected in the 2-cell SM, 21 miRNAs were detected in 8-cell SM, and 77 miRNAs were detected in blastocyst SM (Figure 1). Overlapping the miRNA list from each SM condition allowed for the identification of condition-specific miRNAs and miRNAs shared between 2 or more conditions (Figure 2). Six miRNAs were solely detected in 2-cell SM, in which 2 were upregulated (bta-miR-2421 and bta-miR-2297) and 4 were downregulated in the SM (bta-miR-296-5p, bta-miR-106b, bta-miR-122, and bta-miR-760-5p) (Figure 3). All the miRNAs detected in the 8-cell SM were shared between one or more SM conditions and no miRNAs were unique to 8-cell SM. Of the 77 miRNAs identified in blastocyst SM, 56 miRNAs were exclusive to the SM condition. Fifty-three miRNAs were upregulated (Supplementary Table 1) and 3 miRNAs were downregulated in the media (Figure 4). The remaining detected miRNAs were shared between 2 or more SM conditions.

### Differentially Expressed miRNAs Shared Between 2 or More SM Conditions

**Figure 5:**
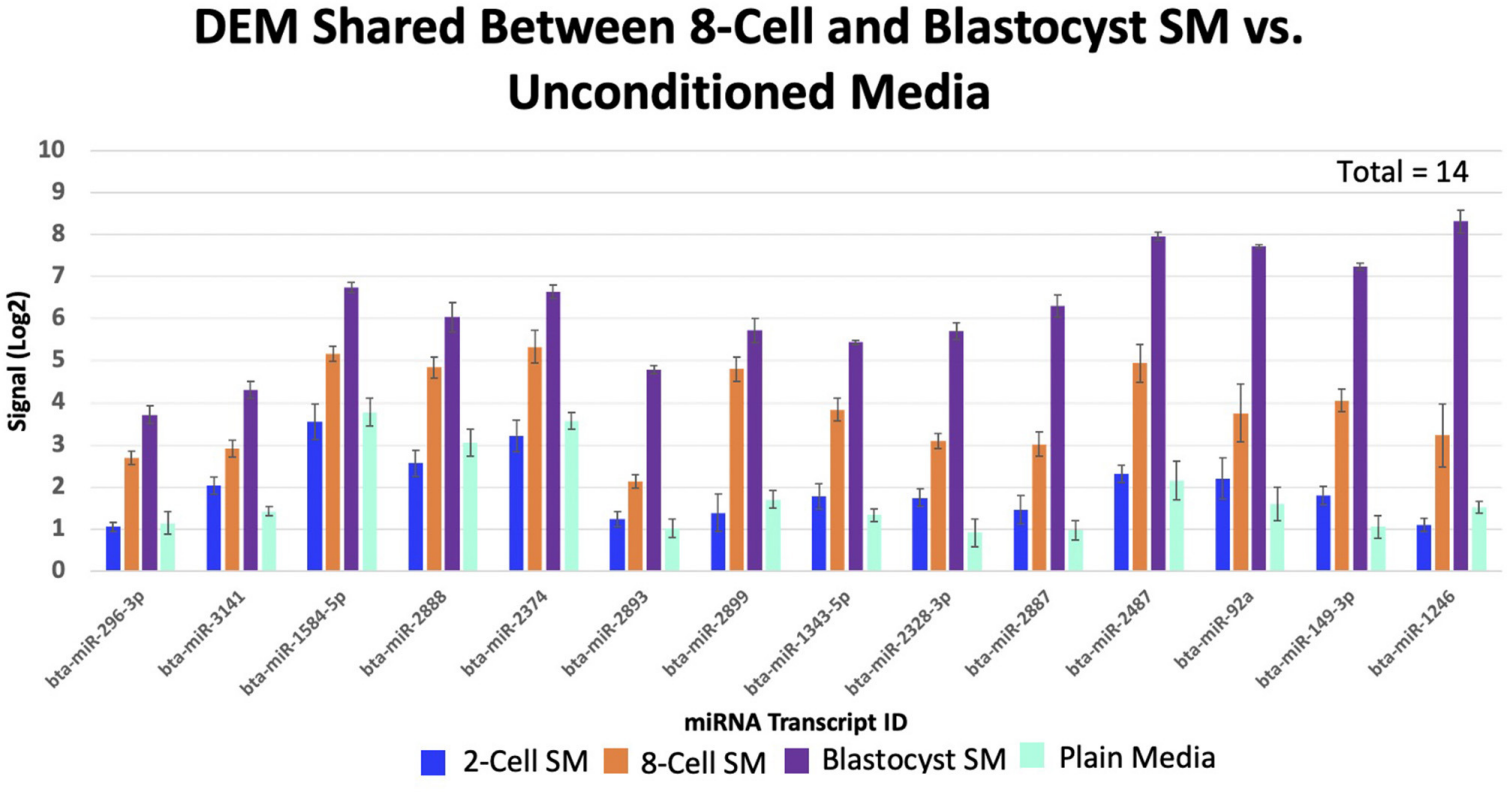
Fourteen miRNAs were significantly shared between 8-cell and blastocyst spent media. All miRNAs increased in levels of up-regulation from the 8-cell to the blastocyst spent media.

**Figure 6:**
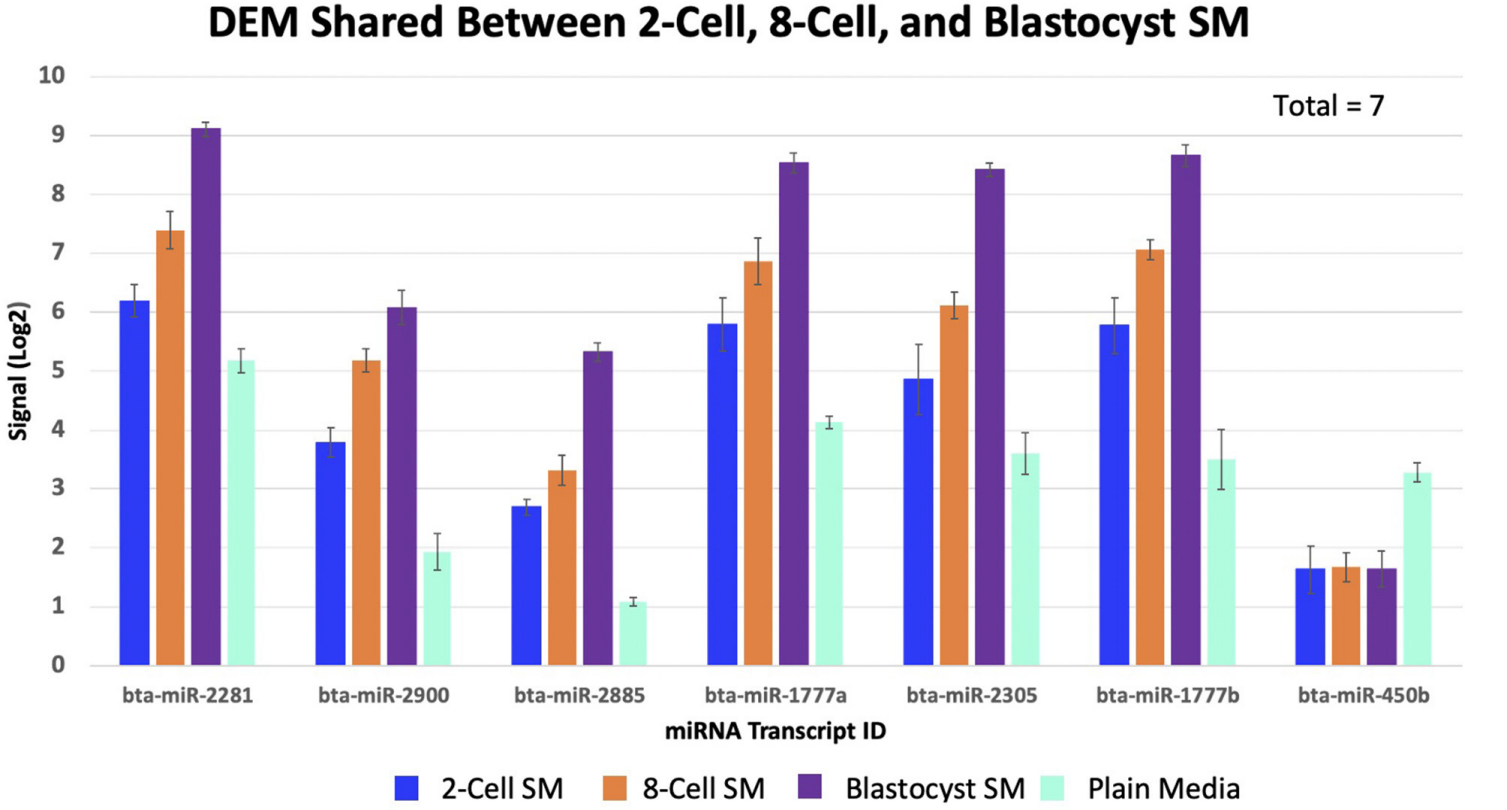
Seven miRNAs were significantly shared between all three spent media conditions. 6 miRNAs increased in levels of up-regulation, while 1 miRNA remained consistently down-regulated (bta-miR-450b).

Overlapping of the gene-list identified miRNAs that were co-detected in 8-cell and blastocyst SM and all 3 SM conditions. Fourteen miRNAs (bta-miR-296-3p, bta-miR-3141, bta-miR-1584-5p, bta-miR-2888, bta-miR-2374, bta-miR-2893, bta-miR-2899, bta-miR-1343-5p, bta-miR-2328-3p, bta-miR-2887, bta-miR-2487, bta-miR-92a, bta-miR-149-3p, and bta-miR-1246) were co-detected in 8-cell and blastocyst SM (Figure 5). All 14 miRNAs were upregulated in comparison to 2 cell SM and unconditioned media. Interestingly, the degree of upregulation increased between 8-cell and blastocyst SM. Seven miRNAs were co-detected in all 3 SM conditions. Six miRNAs were upregulated (bta-miR-2281, bta-miR-2900, bta-miR-2885, bta-miR-1777a, bta-miR-2305, and bta-miR-1777b) (Figure 6) in comparison to unconditioned media and the degree of upregulation increased from one condition to another. One miRNA was consistently downregulated (bta-miR-450b) in comparison to unconditioned media and the degree of downregulation was consistent across all 3 conditions.

### Predictions of miRNA-mRNA Targets for Stage-Specific and Shared Differentially Expressed miRNAs

#### 2-Cell SM

**Figure 7:**
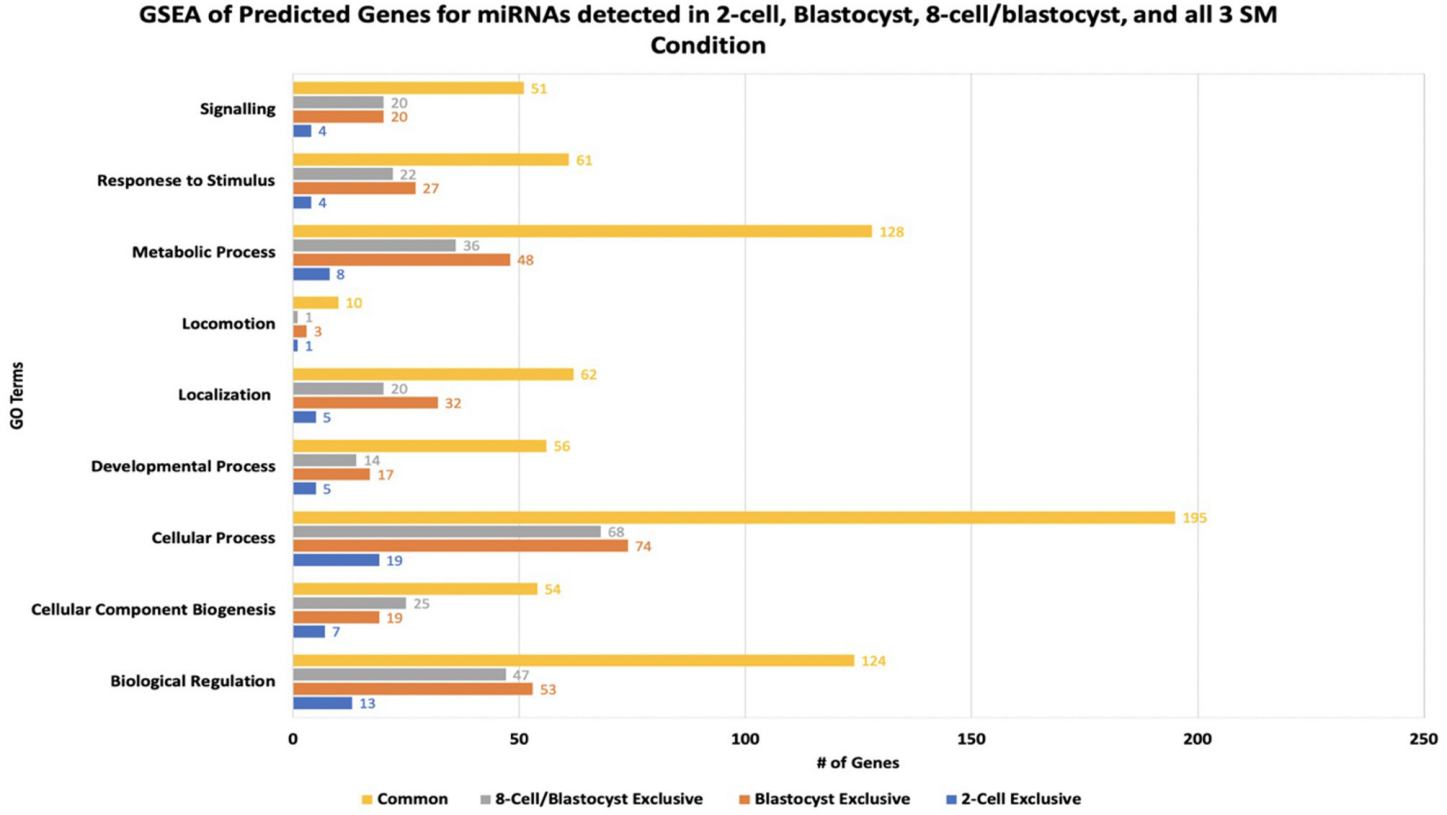
Results of gene-set enrichment analysis of mRNA targets predicted for differentially expressed miRNAs in 2-cell spent media, blastocyst spent media, shared in 8-cell and blastocyst spent media, and common to all 3 spent media conditions. The majority of predicted genes clustered under biological processes involved in cellular process, biological regulation, and metabolic process.

miRNA-mRNA target prediction identified 44 mRNAs as potential targets for the 6 miRNAs identified exclusively to 2-cell SM (Supplementary Table 2). When inputted into PANTHER, 42 genes were associated with the 44 predicted mRNAs, with the majority of genes clustering under cellular process (GO:0009987), biological regulation (GO:0065007), and metabolic process (GO:0008152) (Figure 7). The highly enriched genes were predicted targets of bta-miR-2421 (Table 2).

**Table 2:** miRNA-mRNA targets predicted to have roles in the top 3 biological pathways represented in gene-set enrichment analysis.

**Highly represented miRNAs**		**Genes represented in cellular process, biological regulation, and metabolic process**
2-Cell spent media	bta-miR-2421	TCF4, POU6F2, RUNX1T1, TNRC6C, ONECUT2, TNRC6B, THRB
Blastocyst spent media	bta-miR-7865	MZB1, FOSB, LAMTOR1, WDTC1, THRA
	bta-miR-2295	MAPK8IP2, H1FX, SCRT1, MTA3, EOMES
	bta-miR-3613b	SIK2, USP42, CDK12, GLE1, IKZF4
Shared in 8-cell and blastocyst spent media	bta-miR-1343-5p	TFCP2L1, KCTD17, SIX5, PRX, SERPINE3
	bta-miR-2899	DLX1, MTA1, TBC1D14, HEYL, HCFC1
Common to 2-cell, 8-cell, and blastocyst spent media	bta-miR-2305	PLA2G2F, SMARCC2, SPRY4, CX3CL1, TEAD2, LMX1B, RUNX3, ERF, TBX6, ELK1, SOX15, SPRED2, DAGLA, HNF4A, COMMD7, ZFHX2, RNF144A, KMT2B, MAPK12, FEV, TIMP2, IKZF4, CSDC2, DERL3, CIC, RNPS1, SAMD4B
	bta-miR-2900	FOXJ2, DDA1, PTK2B, HOXA3, CNOT3, MAPK1, SRRM4, NRBP1, PPARD, TRAF3, NODAL, PRKCG, PIAS4, TCF7L2
	bta-miR-1777a	MSI1, CACNG7, TBX5, BRSK1, CRTC1, ZC3H4, TBX10, IGF1R, LEMD2, CTIF, HIF3A, DUSP16, SOX12

#### Blastocyst SM

Due to the high numbers of miRNAs detected in blastocyst SM, only the top 20 upregulated and 3 downregulated miRNAs were considered for miRNA-mRNA target prediction. Of the top 20 upregulated miRNAs, 5 miRNAs (bta-miR-320a, bta-miR-3432, bta-let-7c, bta-miR-191, and bta-miR-378) were excluded from analysis due to predicted genes not meeting the cumulative context score of < −0.75 cutoff or not being found on the TargetScan database. From the remaining 18 miRNAs, a total of 218 mRNAs were identified as possible targets for blastocyst-specific miRNAs (Supplementary Table 3). When inputted into PANTHER, 186 genes were associated with the 218 predicted mRNAs, with the majority of genes clustering under cellular process (GO:0009987), biological regulation (GO:0065007), and metabolic process (GO:0008152) (Figure 7). The highly enriched genes were predicted targets of bta-miR-7865, bta-miR-2295, and bta-miR-3613b (Table 2).

#### 8-Cell and Blastocyst Shared miRNAs

Bta-miR-296-3p and bta-miR-2487 did not meet the cutoff criteria and was not found in the TargetScan database, respectively, thus was excluded from the analysis. Of the remaining 12 miRNAs, a total of 194 mRNAs were identified as potential targets (Supplementary Table 4). When inputted into PANTHER, 168 genes were associated with the 194 predicted mRNAs, with the majority of genes clustering under cellular process (GO:0009987), biological regulation (GO:0065007), and metabolic process (GO:0008152) (Figure 7) The highly enriched genes were predicted targets of bta-miR-1343-5p and bta-miR-2899 (Table 2).

#### miRNAs Common to 2-Cell, 8-Cell, and Blastocyst SM

Bta-miR-2881 was not found in the database, thus was excluded from the overall analysis. Of the remaining 6 miRNAs co-detected in all 3 SM condition, a total of 527 miRNAs were identified as potential targets (Supplementary Table 5). When inputted into PANTHER, 445 genes were associated with the 527 predicted mRNAs, with the majority of genes clustering under cellular process (GO:0009987), biological regulation (GO:0065007), and metabolic process (GO:0008152) (Figure 7). The highly enriched genes were predicted targets of bta-miR-2305, bta-miR-2900, and bta-miR-1777a (Table 2).

Overall, GSEA analysis revealed that the majority of the miRNAs detected in the 4 conditions (2-cell SM, 8-cell SM, 8-cell/blastocyst SM, and common in all 3 SM) had predicted mRNA targets involved in cellular process, biological regulation, and metabolic process. For 2-cell SM, bta-miR-2421 and bta-miR-760-5p mRNA targets were over-represented after enrichment. In blastocyst SM, bta-miR-7865, bta-miR-2295, and bta-miR-3613b mRNA targets were highlighted most during GSEA analysis. For miRNA shared between two or more SM conditions, bta-miR-1343-5p, bta-miR-2899, bta-miR-2888, bta-miR-2305, bta-miR-2900, and bta-miR-1777a mRNA targets were highly represented in the 3 biological processes highlighted in PANTHER.

### Annotated Roles of Differentially Expressed miRNAs in Literature

From the 111 miRNAs identified across 3 SM conditions, 32 miRNAs have been previously annotated in literature. 18 miRNAs have been identified in embryo related studies, 14 miRNAs in cancer-related studies, and 5 miRNAs in both embryo and cancer related studies. miRNAs (Table 3). It is important to note that all previously annotated miRNAs were exclusively detected in blastocyst SM.

**Table 3:** List of miRNAs detected in all 3 spent media conditions previously annotated in literature.

**Study-Type**		**miRNA**
Embryo-Related	Spent media profiling	miR-24-3p, miR-191, miR-2887
	Role in implantation	miR-320a^*^, let-7b, miR-23b-3p^*^, miR-23a, miR-3141, miR-92a^*^, miR-1246
	Role in differentiation	miR-371, miR-296-5p/3p^*^, miR-106b^*^, miR-125b
	Found in germ cells	miR-3432, miR-2487, miR-2885, miR-1777b
Cancer-Related	Onco-genic	miR-106b^*^, miR-760-5p, miR-371, let-7d, miR-222, miR-378, miR-1343-5p, miR-92a^*^
	Tumor-suppressive	miR-296-5p/3p^*^, miR-320a^*^, miR-320b, miR-23b-3p^*^, miR-342, let-7c, let-7d, miR-191, miR-149-3p, miR-1246, miR-450b

*The majority (18 miRNAs) have been profiled in embryo-based studies, while 14 miRNAs were explored in cancer-related studies. 5 miRNAs were cited in both embryo and cancer related studies*.

* *Found in both embryo and cancer relate studies*.

## Discussion

To the best of our knowledge, this study was the first to characterize miRNAs in the SM throughout the pre-implantation period. We were able to identify miRNAs in the SM at early, mid, and late stages of development. The most diverse expression of miRNAs was detected in blastocyst SM. This finding is consistent with the works of other researchers (15–17, 21) and is largely attributable to the genetic, molecular, and cellular changes occurring during this stage of embryo development. An embryo during this stage rapidly proliferates and differentiates to form the ICM and trophectoderm. Differentiation is a highly choreograph events that requires strict modulation of gene expression. Potentially, miRNAs play a role in synchronizing the events necessary for normal blastocyst development. Therefore, miRNA expression may increase during this stage of development, which is reflected in the SM.

Another explanation to the high numbers of miRNAs detected in blastocyst SM is due to the high cell numbers present at this stage of development. Prior to compaction and blastocyst formation, a growing embryo is made up of relatively few cells, enough to be distinguishable under stereomicroscope. With such few cells prior to compaction, it is believed that miRNA concentrations are far too low to be detected with current assays. In a recent study, it was determined that the majority of miRNAs detected in blastocyst SM was trophectoderm derived (22). Thus, this may contribute to the low numbers of miRNAs detected in earlier stages as the trophectoderm only forms at later stages of embryo development. Interestingly, the authors also postulated that the blastocyst secretes miRNAs as a method of paracrine communication with the endometrium (22). The diverse population of miRNAs secreted by trophectoderm cells may be up taken by endometrial cells. Once inside, the miRNAs may modulate gene expression to either favor or prevent the implantation of a blastocyst. Therefore, the diverse expression of miRNAs in blastocyst SM may serve a functional role in implantation and/or be a direct by-product of the high cell numbers present at this stage of development.

One finding that was unique in this study was the detection of miRNAs in cleavage stage embryos. Specifically, we were able to detect 6 miRNAs exclusive to 2-cell SM. Moreover, 2-cell SM was the only condition examined that had more miRNAs down-regulated (4 miRNAs) than up-regulated (2 miRNAs). These findings are interesting because this is the first report of miRNAs being detected in 2-cell SM. The wide coverage of miRNAs available on the microarray may have allowed for the detection of miRNAs. The genechip miRNA 4.0 array used for this study had over 46,228 probes comprising of 7,815 probe sets from 71 different species. Since the majority of the miRNAs identified in 2-cell SM and 8-cell SM are ones not previously annotated in embryo-related work, it is possible that the assays previous researchers used did not contain the probes present in our study. Most studies either examined a select few candidate miRNAs using qPCR or a wider population with qPCR array. Although both methods allow for higher sensitivity, lower sample input, and lower false detection rates, both approaches have fewer probe sets than the ones used in this study.

In addition to having more probe-sets, group culturing of embryo may have impacted miRNA detection. Prior studies examining miRNAs throughout the pre-implantation period performed either single-embryo culture or had a lower ratio of embryos to culture media volume. These culturing conditions allowed researchers to discern the miRNAs in the SM to specific embryos. However, as mentioned before, low cell numbers contribute to the lack of miRNAs detected in media. Therefore, previous researcher was only able to detect miRNAs from blastocyst conditioned media, whereby cell numbers are higher. In this study, embryos were cultured in groups of 30 in 30 μl of IVC media. The higher concentration of embryos in the media may have compensated for the few cells available to secrete and/or uptake miRNAs in the media. Therefore, the diversity in the probe-sets present in the array used, in conjunction with higher embryos concentrations may have allowed for the detection of miRNAs in SM cultured with cleavage staged embryos. To this regard, collecting SM from group cultured embryos does not allow for the detection of embryo-specific miRNAs. Therefore, assays such as digital drop PCR, may be used in future SM profiling experiments as its low sample input volume and high sensitivity may suffice in detecting miRNAs in single embryo culture (23).

Another interesting result from the miRNAs detected in 2-cell SM is that it is the only condition where more down-regulated miRNAs were detected in comparison to upregulated miRNAs. This indicates that the 2-cell embryo may be capable of up-taking miRNAs from the extracellular environment. Although undocumented in cleavage stage embryos, previous research has demonstrated that morula-stage embryos are capable of up-taking miRNAs, with subsequent effects on gene expression affecting blastocyst development. Our findings suggest that embryos may be able to up take miRNAs throughout the preimplantation period. This highlights a potential area for the development of therapeutics, whereby certain miRNAs can be supplemented into culture media to modulate gene expression that favors normal embryo development.

Conversely, our findings also highlight the gap in knowledge regarding the native miRNA population found in commercially available culture media. Presently, most culture medias, including ones used for the *in-vitro* maturation, fertilization, and culture of embryos in this study, use serum as a means of improving blastocyst yield. Serum contains various growth factors, adhesion factors, trace hormones, lipids, and minerals essential for the *in-vitro* culture of embryos. Since miRNAs can be found in a wide array of biofluids, it can be postulated that the serum used in culture media contain miRNAs. Currently, the miRNA profile of culture medias are not known and/or disclosed, however, research have shown that miRNAs are present in plain culture media. Instead of modulating genes to stimulate growth, miRNAs may work to negatively inhibit growth and development as observed by Kropp et al. (2015). Since our findings suggest that embryos may be capable of up-taking miRNAs as early as the 2-cell stage, a detailed characterization of miRNAs in culture media may serve to improve the efficiency of *in-vitro* production systems. miRNAs known to negatively affect growth may be removed with the subsequent supplementation of miRNAs known to stimulate development. With the widespread availability of commercially available serum free culture medias, the groups examined in this study can also be conducted using serum-free culture systems. Without the presence of endogenous miRNAs in culture systems, this may identify additional miRNAs secreted by embryos and/or determine the true source of miRNAs detected in the SM from this study.

Focusing on our findings in 8-cell SM, it was determined that 21 miRNAs were detected with no miRNAs exclusively found in the media. It is important to note that EGA occurs during the 8-cell stage in bovine embryos. Perhaps that lack of miRNAs exclusive to 8-cell SM may be related to this developmental event. EGA is the period in embryo development when maternally inherited transcripts are degraded, and transcription of embryonic genome begins (24). miRNAs, on the other hand, serve to primarily inhibit gene expression. Tesfaye et al. (14) do report that intracellular miRNA expression in embryos change dynamically. Some miRNAs are highly expressed in early and late stages of development, while being absent during EGA. Therefore, our findings suggest that miRNA expression may temporally dampened during the 8-cell stage to allow for the events of EGA to proceed.

Although no miRNAs were exclusively detected in 8 cell SM, 7 of the 21 miRNAs were shared between across all 3 SM conditions. This is a significant finding as this is the first instance of miRNAs being demonstrated to have consistent expression in the SM throughout the preimplantation period. Moreover, 6 miRNAs increased, and 1 miRNA stayed consistently downregulated in expression from one condition to another. Therefore, it may indicate that these miRNAs may play a housekeeping role throughout embryonic development. GSEA analysis did reveal that bta-miR-2305, bta-miR-2900, and bta-miR-1777a mRNA targets were overrepresented in pathways pertaining to cellular process, biological regulation, and metabolic process. Regulation of these pathways are all necessary requirements for embryo development. Perhaps, the promiscuous nature of miRNAs may allow certain miRNAs to be expressed throughout development but regulate different genes in a stage specific manner. Due to the detection of these miRNAs in 2-cell SM, it does present a possibility that these 7 shared miRNAs may be of maternal origin. Evidence suggest that the majority miRNAs expressed in early embryo development are oocyte derived. Therefore, future experiments may profile SM conditioned with mature oocytes for miRNAs. Perhaps, the potential housekeeping roles these miRNAs play in early embryo development may be maternally derived, thus strengthening the relationship of oocyte quality and downstream overall embryo quality.

It is important to note that bta-miR-450b was the only miRNA consistently downregulated across all conditions. Aside from stage specific up take of miRNAs highlighted from our 2-cell SM result, this finding indicates that certain miRNAs may be up-taken throughout the preimplantation period. Thus, this reinforces the idea of embryo having the ability to interact with its environment. Expression of bta-miR-450b was higher in plain media in comparison to all SM condition, suggesting that this miRNA is native to the commercial media used in the study. There is little known regarding bta-miR-450b in embryo development, but a predicted mRNA target is CAM2KN1 (calcium/calmodulin-dependent protein kinase II). In previous studies, it has been demonstrated that CAM2KN1 is an oncogene present in prostate cancer tissue (25). When CAM2KN1 expression was reduced, cell proliferation was impaired and apoptosis was increased (25). Therefore, it is possible that up take of bta-miR-450b in embryos may cause a subsequent decrease in expression of CAM2KN1 or other oncogenes, thereby impairing cell proliferation. Although this has not been explored, it once again highlights the need to profile the miRNA expression across commercially available medias.

Aside from the 7 miRNAs expressed across all 3 SM conditions, our study also detected 14 miRNAs that increased in expression in the SM from the 8-cell-to the blastocyst stage of development. It is known that specific miRNAs are expressed in the embryo from the period of EGA to blastocyst formation, however, no study have been able to demonstrate these changes in the extracellular environment. Therefore, the 14 miRNAs that we identified may play a role in initiating and facilitating an embryos development post EGA. Although none of the miRNAs co-detected in 8-cell and blastocyst SM have been annotated in previous SM and embryo studies, GSEA revealed that predicted mRNA targets impact pathways regulating proliferation, differentiation, and metabolism.

From a broad perspective, the vast majority of miRNAs detected in this study have not been previously annotated in literature, thus their functions are widely unknown. However, literature search did reveal that 32 of the 56 miRNAs detected in blastocyst SM have been previously explored in research. miR-24-3p, miR-191, and miR-2887 have been detected in SM cultured with embryos that failed to progress to the blastocyst stage of development (17). Moreover, miR-320a, let-7b, miR-23b-3p, miR-23a, miR-3141, miR-92a, miR-1246 have been profiled in SM conditioned with embryos that failed to implant (22). Although our study did not separate embryos based on blastocyst outcome and implantation, it appears that the miRNAs detected in blastocyst SM may be derived from poor quality blastocyst and/or ones that arrested. It has been observed that poorer quality embryos have a more dynamic expression of proteins and metabolites (26). Potentially, this dynamic expression may also translate with miRNAs in SM.

It should also be noted that a cohort of blastocyst exclusive miRNAs have been previously annotated in cancer-related studies. Specifically, some blastocyst specific miRNAs have been cited to have either an oncogenic and/or tumor suppressive role in various tissues such as kidney, liver, prostate, and ovary. It is well-established that parallels exist in the biological pathways used in embryogenesis and tumorigenesis. Molecular cues to govern morphological change, differentiation, proliferation, and invasion are all used by the pre-implantation embryo to establish pregnancy. These same processes are used by tumor cells to enhance growth, recruit cells, and coordinate spread to distant tissues. Therefore, connecting the findings of oncogenic studies may be invaluable in unlocking the roles of miRNAs in embryogenesis.

Overall, this study was able to detect miRNAs in the SM across the pre-implantation period. Cross-referencing of miRNAs from each condition allowed for the identification of stage-specific miRNAs and those shared across 2 or more SM conditions. All of the miRNAs identified have gene targets relating to pathways regulating cellular processes, biological regulation, and metabolism. Although the roles of the miRNAs identified are mostly unknown, those that have been identified suggest that miRNAs are indicative of intrinsic embryonic physiology. Future research should focus on qualitatively validating characterized miRNAs within the SM and intracellularly. From these findings, miRNA-mRNA target knockdowns can be performed to elucidate the roles miRNAs play in early embryo development.

## Data Availability Statement

The datasets presented in this study can be found in online repositories. The names of the repository/repositories and accession number(s) can be found at: https://www.ncbi.nlm.nih.gov/geo/query/acc.cgi?acc=GSE168551.

## Author Contributions

PD and PM conceived, developed, and planned the experiments. PD performed all the experiments and including sample preparation and data analysis. The manuscript was written by PD with the support and guidance of PM. PM supervised the project.

## Conflict of Interest

The authors declare that the research was conducted in the absence of any commercial or financial relationships that could be construed as a potential conflict of interest.
