# Supplementary material for: Does miRNA Expression in the Spent Media Change During Early Embryo Development?
Source: Front Vet Sci. 2021 Apr 8;8:658968. doi: 10.3389/fvets.2021.658968 (PMC8060439; doi:10.3389/fvets.2021.658968)
Supplement: Supplementary file 1 [file Data_Sheet_1.PDF]

Supplementary Tables

S. Table 1. DEM in blastocyst SM

miRNAs	Fold-Change	miRNAs	Fold-Change	miRNAs	Fold-Change
bta-miR-371	77.52	bta-miR-17-5p	5.55	bta-miR-346	2.93
bta-miR-320a	43.52	bta-miR-361	5.38	bta-miR-6535	2.8
bta-miR-3432	29.92	bta-miR-2407	5.14	bta-miR-16a	2.59
bta-let-7b	24.94	bta-miR-125a	5.04	bta-miR-30a-5p	2.58
bta-miR-24-3p	19.02	bta-miR-2455	4.99	bta-miR-744	2.4
bta-miR-23b-3p	15.97	bta-miR-423-5p	4.97	bta-miR-151-5p	2.39
bta-miR-7865	14.99	bta-let-7a-5p	4.74	bta-miR-182	2.37
bta-miR-342	14.38	bta-miR-30d	4.44	bta-miR-664	2.2
bta-miR-2402	12.83	bta-miR-2442	4.4	bta-miR-2324	2.2
bta-miR-320b	10.27	bta-miR-6529	4.3	bta-miR-2348	2.13
bta-let-7c	10.27	bta-miR-2904	4.07	bta-miR-155	2.13
bta-miR-23a	10.08	bta-miR-26a	3.95	bta-miR-2288	2.04
bta-miR-2295	8.48	bta-miR-193a-5p	3.93	bta-miR-669	2.01
bta-let-7d	8.36	bta-miR-2392	3.81	bta-miR-2457	-2.24
bta-miR-222	8.26	bta-miR-2426	3.7	bta-miR-2287	-2.74
bta-miR-191	7.54	bta-miR-221	3.39	bta-miR-3613b	-3.42
bta-miR-2436-5p	6.92	bta-miR-2413	3.17		
bta-miR-2412	6.5	bta-miR-2428	3.16		
bta-miR-125b	5.78	bta-miR-2309	3.12		
bta-miR-378	5.59	bta-miR-20a	3		

S. Table 2 Predicted mRNA targets of DEM in 2-cell SM

miRNA	Predicted mRNAs	Cumulative Context Score	miRNAs	Predicted mRNAs	Cumulative Context Score
bta-miR-2421	XKR4	-2.17	bta-miR-122	GYS1	-0.96
bta-miR-2421	NFIA	-1.79	bta-miR-760-5p	CD300LB	-1.29
bta-miR-2421	ONECUT2	-1.5	bta-miR-760-5p	GORASP1	-1.27
bta-miR-2421	TCF4	-1.21	bta-miR-760-5p	AC016722.1	-0.99
bta-miR-2421	ELAVL4	-1.14	bta-miR-760-5p	CT62	-0.98
bta-miR-2421	NFIB	-1.09	bta-miR-760-5p	GLIPR2	-0.93
bta-miR-2421	THRB	-1	bta-miR-760-5p	SLC4A11	-0.9
bta-miR-2421	GABRB3	-1	bta-miR-760-5p	RHOU	-0.87
bta-miR-2421	POU6F2	-0.99	bta-miR-760-5p	OR5AU1	-0.85
bta-miR-2421	TNRC6C	-0.92	bta-miR-760-5p	PNKD	-0.84
bta-miR-2421	IGIP	-0.91	bta-miR-760-5p	OR1L3	-0.84

bta-miR-2421	TNRC6B	-0.87	bta-miR-760-5p	DOCK1	-0.82
bta-miR-2421	GLIPR1L1	-0.82	bta-miR-760-5p	PRPH2	-0.81
bta-miR-2421	CYLC2	-0.81	bta-miR-760-5p	DMKN	-0.78
bta-miR-2421	RUNX1T1	-0.75	bta-miR-760-5p	DNAJC15	-0.77
bta-miR-2297	SRCAP	-1	bta-miR-760-5p	LRP11	-0.76
bta-miR-2297	G3BP1	-1	bta-miR-760-5p	POSTN	-0.75
bta-miR-2297	C17orf58	-0.93	bta-miR-760-5p	CYHR1	-0.75
bta-miR-2297	DCAF4L2	-0.84			
bta-miR-2297	JTB	-0.82			
bta-miR-2297	TSC22D3	-0.8			
bta-miR-2297	C15orf60	-0.77			
bta-miR-296-5p	FAU	-0.93			
bta-miR-296-5p	MS4A13	-0.84			
bta-miR-296-5p	APOE	-0.76			
bta-miR-296-5p	BET1L	-0.76			

S. Table 3. Predicted mRNA targets of DEM in blastocyst SM

miRNA	Predicted mRNAs	Cumulative Context Score	miRNAs	Predicted mRNAs	Cumulative Context Score
bta-miR-371	BOD1L2	-1.17	bta-miR-2295	SCRT1	-0.91
bta-let-7b	ST8SIA1	-0.8	bta-miR-2295	TMEM109	-0.88
bta-miR-24-3p	STRADB	-0.85	bta-miR-2295	CERK	-0.87
bta-miR-24-3p	TCF7	-0.84	bta-miR-2295	OXLD1	-0.86
bta-miR-24-3p	C12orf43	-0.84	bta-miR-2295	CAMTA2	-0.85
bta-miR-24-3p	ENTPD6	-0.84	bta-miR-2295	PTPN7	-0.85
bta-miR-24-3p	LSM10	-0.79	bta-miR-2295	XKR7	-0.84
bta-miR-24-3p	SNN	-0.78	bta-miR-2295	H1FX	-0.84
bta-miR-24-3p	GBA2	-0.76	bta-miR-2295	MYPOP	-0.84
bta-miR-24-3p	SLCO2B1	-0.75	bta-miR-2295	ZNF385A	-0.83
bta-miR-23b-3p	SS18L2	-1.09	bta-miR-2295	MAPK8IP2	-0.83
bta-miR-23b-3p	ELF5	-0.81	bta-miR-2295	SLC27A4	-0.81
bta-miR-7865	PRRT2	-2.49	bta-miR-2295	GPR68	-0.8
bta-miR-7865	SYNGR1	-1.32	bta-miR-2295	CDK2AP2	-0.8
bta-miR-7865	PIANP	-1.17	bta-miR-2295	ACKR2	-0.8
bta-miR-7865	NFIC	-1.16	bta-miR-2295	SYNGR4	-0.79
bta-miR-7865	C12orf36	-1.15	bta-miR-2295	LTB	-0.78
bta-miR-7865	TSPAN18	-1.12	bta-miR-2295	CSNK2B-LY6G5B-1181	-0.78
bta-miR-7865	SHISA6	-1.08	bta-miR-2295	POLR2J2	-0.77

bta-miR-7865	RNF165	-1.08	bta-miR-2295	BEST4	-0.77
bta-miR-7865	BOD1L2	-1.07	bta-miR-2295	FXYD5	-0.76
bta-miR-7865	CREB3L2	-1.04	bta-miR-2295	KCNK3	-0.76
bta-miR-7865	C1orf68	-1.03	bta-miR-2295	PPAPDC1A	-0.76
bta-miR-7865	AIF1L	-1	bta-miR-2295	AQP7	-0.76
bta-miR-7865	GORASP1	-0.99	bta-miR-2295	SLC38A10	-0.75
bta-miR-7865	SYNGR4	-0.99	bta-miR-2295	KIAA0930	-0.75
bta-miR-7865	PKLR	-0.96	bta-miR-2295	EOMES	-0.75
bta-miR-7865	UPK2	-0.94	bta-miR-2295	APOE	-0.75
bta-miR-7865	MARVELD1	-0.92	bta-miR-2295	LAMTOR4	-0.75
bta-miR-7865	OLIG2	-0.9	bta-let-7d	HMGA2	-2.74
bta-miR-7865	KRT80	-0.89	bta-let-7d	FIGN	-1.53
bta-miR-7865	MS4A8	-0.86	bta-let-7d	ARID3B	-1.42
bta-miR-7865	DCX	-0.85	bta-let-7d	LIN28B	-1.41
bta-miR-7865	ADAM19	-0.85	bta-let-7d	TRIM71	-1.27
bta-miR-7865	ZNF226	-0.85	bta-let-7d	POLR2J2	-1.03
bta-miR-7865	DCTN3	-0.84	bta-let-7d	LIN28A	-0.92
bta-miR-7865	MYADM	-0.84	bta-let-7d	USP44	-0.91
bta-miR-7865	ATP6V0E2	-0.84	bta-let-7d	FZD3	-0.89
bta-miR-7865	THRSP	-0.84	bta-let-7d	IGDCC3	-0.87
bta-miR-7865	HNRNPH2	-0.84	bta-let-7d	AC140061.12	-0.87
bta-miR-7865	CYP8B1	-0.83	bta-let-7d	IGF2BP1	-0.86
bta-miR-7865	GUCA1A	-0.82	bta-let-7d	YOD1	-0.84
bta-miR-7865	THRA	-0.82	bta-let-7d	VSTM5	-0.84
bta-miR-7865	LAMTOR4	-0.82	bta-let-7d	ZBTB8B	-0.84
bta-miR-7865	PRR24	-0.81	bta-let-7d	PPP1R15B	-0.8
bta-miR-7865	AKAP5	-0.81	bta-let-7d	FAM222B	-0.8
bta-miR-7865	MT-ND4L	-0.81	bta-let-7d	CDK8	-0.8
bta-miR-7865	FOSB	-0.81	bta-let-7d	NGF	-0.8
bta-miR-7865	SST	-0.81	bta-let-7d	CCL7	-0.78
bta-miR-7865	HOXC8	-0.8	bta-let-7d	HAND1	-0.75
bta-miR-7865	SLC7A8	-0.8	bta-miR-222	PVRL1	-1.63
bta-miR-7865	MAFG	-0.8	bta-miR-222	GABRA1	-1.11
bta-miR-7865	PPME1	-0.8	bta-miR-222	CDKN1B	-1.04
bta-miR-7865	KCNJ10	-0.8	bta-miR-222	PGAP1	-0.99
bta-miR-7865	MZB1	-0.8	bta-miR-2436-5p	MYL6B	-1.18
bta-miR-7865	SPRYD3	-0.79	bta-miR-2436-5p	DGCR2	-1.15
bta-miR-7865	URM1	-0.78	bta-miR-2436-5p	ZNF282	-1.08
bta-miR-7865	GIPC1	-0.78	bta-miR-2436-5p	MZF1	-1.07
bta-miR-7865	RTBDN	-0.78	bta-miR-2436-5p	REPIN1	-0.93

bta-miR-7865	RASL10B	-0.78	bta-miR-2436-5p	CYP2F1	-0.86
bta-miR-7865	WFDC2	-0.77	bta-miR-2436-5p	CNPY3	-0.84
bta-miR-7865	LAMTOR1	-0.77	bta-miR-2436-5p	KAAG1	-0.76
bta-miR-7865	SPR	-0.77	bta-miR-2436-5p	CCL4L2	-0.76
bta-miR-7865	WDTC1	-0.76	bta-miR-2412	PML	-1.17
bta-miR-7865	HNRNPUL1	-0.76	bta-miR-2412	COX6B2	-1.09
bta-miR-7865	AK4	-0.76	bta-miR-2412	TNFSF13	-1.04
bta-miR-7865	NHLH1	-0.76	bta-miR-2412	C15orf32	-1.03
bta-miR-7865	NRSN2	-0.75	bta-miR-2412	WNT4	-0.97
bta-miR-342	FAM53C	-0.83	bta-miR-2412	CTD-3203P2.2	-0.91
bta-miR-2402	C1orf134	-0.98	bta-miR-2412	SNX32	-0.85
bta-miR-2402	ZMAT3	-0.87	bta-miR-2412	TNFSF12-TNFSF13	-0.85
bta-miR-2402	GDI2	-0.86	bta-miR-2412	SLC34A2	-0.81
bta-miR-2402	MRPL32	-0.84	bta-miR-2412	C1QTNF6	-0.78
bta-miR-2402	AC117834.1	-0.81	bta-miR-2412	KXD1	-0.75
bta-miR-2402	C19orf53	-0.77	bta-miR-125b	RNPEPL1	-0.79
bta-miR-320b	GIPC3	-4.41	bta-miR-125b	DRAM2	-0.79
bta-miR-320b	HIPK2	-1.62	bta-miR-125b	ARID3B	-0.75
bta-miR-320b	NFIC	-1.45	bta-miR-2457	IQCJ	-1.18
bta-miR-320b	SPN	-1.2	bta-miR-2457	CRYGC	-1.08
bta-miR-320b	FAM43B	-1.05	bta-miR-2457	SPIN3	-0.8
bta-miR-320b	ONECUT3	-0.97	bta-miR-2287	PRAF2	-0.9
bta-miR-320b	POLR2J2	-0.93	bta-miR-2287	WDR45	-0.82
bta-miR-320b	HIVEP3	-0.92	bta-miR-2287	AF196779.12	-0.82
bta-miR-320b	C3orf72	-0.91	bta-miR-2287	C15orf48	-0.75
bta-miR-320b	TGM2	-0.89	bta-miR-2287	ZNF397	-0.75
bta-miR-320b	RPL39	-0.87	bta-miR-3613b	LGI2	-1
bta-miR-320b	ST3GAL3	-0.87	bta-miR-3613b	DEDD	-1
bta-miR-320b	AL049747.1	-0.83	bta-miR-3613b	KDELR2	-1
bta-miR-320b	BET1L	-0.8	bta-miR-3613b	MRRF	-1
bta-miR-320b	KCNK3	-0.79	bta-miR-3613b	GOLT1B	-1
bta-miR-320b	POP5	-0.77	bta-miR-3613b	ZFP37	-1
bta-miR-320b	SDK2	-0.77	bta-miR-3613b	USP38	-1
bta-miR-320b	C10orf53	-0.77	bta-miR-3613b	IKZF4	-1
bta-miR-320b	NPTXR	-0.76	bta-miR-3613b	SNX30	-1
bta-miR-320b	HSFX1	-0.76	bta-miR-3613b	USP42	-1
bta-miR-320b	CHTF8	-0.76	bta-miR-3613b	CEP128	-1
bta-miR-320b	TFRC	-0.75	bta-miR-3613b	CCDC132	-1
bta-miR-23a	ZNF655	-1.15	bta-miR-3613b	SIK2	-1
bta-miR-23a	ACVR1C	-0.92	bta-miR-3613b	RHOQ	-1

bta-miR-23a	TFRC	-0.77	bta-miR-3613b	AGAP2	-1
bta-miR-23a	PNRC2	-0.76	bta-miR-3613b	RBM25	-1
bta-miR-23a	PKP4	-0.75	bta-miR-3613b	GAPVD1	-1
bta-miR-2295	AC006372.1	-1.65	bta-miR-3613b	GAS7	-1
bta-miR-2295	NAPA	-1.45	bta-miR-3613b	CPD	-1
bta-miR-2295	MTA3	-1.45	bta-miR-3613b	HSD17B12	-1
bta-miR-2295	MVB12B	-1.16	bta-miR-3613b	TMBIM6	-1
bta-miR-2295	CCDC69	-1.15	bta-miR-3613b	GLE1	-1
bta-miR-2295	RP11-429E11.3	-1.03	bta-miR-3613b	MFN2	-1
bta-miR-2295	AL391421.1	-1.02	bta-miR-3613b	CDK12	-1
bta-miR-2295	AIDA	-1	bta-miR-3613b	ARHGAP35	-1
bta-miR-2295	THRA	-0.99	bta-miR-3613b	FAM126A	-1
bta-miR-2295	SORCS2	-0.94	bta-miR-3613b	ZIC5	-1
bta-miR-2295	RAB35	-0.92	bta-miR-3613b	KPNA6	-1
bta-miR-2295	FMNL1	-0.92	bta-miR-3613b	LONRF2	-1

S. Table 4. Predicted mRNA targets of DEM in 8-cell and blastocyst SM

miRNA	Predicted mRNAs	Cumulative Context Score	miRNAs	Predicted mRNAs	Cumulative Context Score
bta-miR-2899	PLXNA1	-2.04	bta-miR-3141	DMTN	-0.86
bta-miR-2899	CMIP	-1.71	bta-miR-2899	CLCN2	-0.86
bta-miR-1584-5p	CPLX2	-1.68	bta-miR-2899	TTYH3	-0.86
bta-miR-1584-5p	LDHAL6B	-1.56	bta-miR-1343-5p	SPSB1	-0.86
bta-miR-2888	TNS1	-1.51	bta-miR-1343-5p	TFCP2L1	-0.86
bta-miR-2899	PACSIN1	-1.51	bta-miR-1343-5p	TFEB	-0.86
bta-miR-1343-5p	PRX	-1.51	bta-miR-1343-5p	FHL3	-0.86
bta-miR-2899	NAT8L	-1.5	bta-miR-2887	TTYH3	-0.86
bta-miR-2899	ARC	-1.49	bta-miR-3141	RAB8A	-0.85
bta-miR-1343-5p	KIAA0513	-1.48	bta-miR-2888	C2orf66	-0.85
bta-miR-2899	GAS8	-1.47	bta-miR-2899	AXIN1	-0.85
bta-miR-2899	BRSK2	-1.42	bta-miR-1343-5p	SIPA1L3	-0.85
bta-miR-1584-5p	GIPC3	-1.35	bta-miR-2899	PHKG2	-0.84
bta-miR-2899	FBXL16	-1.34	bta-miR-1343-5p	LDLRAP1	-0.84
bta-miR-1584-5p	RP11-131H24.4	-1.32	bta-miR-2888	CD3E	-0.83
bta-miR-1343-5p	KSR2	-1.29	bta-miR-2374	C9orf171	-0.83
bta-miR-2899	OR10S1	-1.27	bta-miR-2899	MXD4	-0.83
bta-miR-1343-5p	RAB37	-1.27	bta-miR-2899	HCFC1	-0.83
bta-miR-3141	MS4A15	-1.24	bta-miR-1343-5p	KCTD17	-0.83

bta-miR-1343-5p	LY6G6C	-1.24	bta-miR-1343-5p	SIX5	-0.83
bta-miR-2899	ADAMTS13	-1.23	bta-miR-2328-3p	IPO11	-0.83
bta-miR-2899	MAFK	-1.22	bta-miR-1584-5p	P2RX6	-0.82
bta-miR-1343-5p	EHD2	-1.22	bta-miR-1584-5p	SPINT1	-0.82
bta-miR-2899	PAM16	-1.2	bta-miR-2374	IQSEC3	-0.82
bta-miR-2899	SYNGR3	-1.18	bta-miR-2374	WDR48	-0.82
bta-miR-2374	G6PC3	-1.17	bta-miR-2899	PHOX2B	-0.82
bta-miR-2888	FAM222B	-1.15	bta-miR-2899	SYT8	-0.82
bta-miR-2328-3p	C6orf163	-1.15	bta-miR-2899	ZNF385A	-0.82
bta-miR-2899	ATP6V0C	-1.14	bta-miR-1343-5p	KB-1507C5.2	-0.82
bta-miR-2899	RCVRN	-1.14	bta-miR-1343-5p	MVB12B	-0.82
bta-miR-1343-5p	C17orf103	-1.13	bta-miR-1584-5p	MAPT	-0.81
bta-miR-2893	RP11-422N16.3	-1.11	bta-miR-2899	RNF166	-0.81
bta-miR-2899	RAX	-1.11	bta-miR-2899	THY1	-0.81
bta-miR-1246	GSG1L	-1.11	bta-miR-2328-3p	ONECUT3	-0.81
bta-miR-2899	ZDHHC8	-1.1	bta-miR-3141	TTYH3	-0.8
bta-miR-2899	PAX2	-1.1	bta-miR-3141	SSR1	-0.8
bta-miR-1343-5p	GAS8	-1.1	bta-miR-3141	RTL1	-0.8
bta-miR-2374	ZCCHC24	-1.09	bta-miR-2888	CPLX2	-0.8
bta-miR-1246	TMPRSS11A	-1.07	bta-miR-2888	PFN1	-0.8
bta-miR-2899	UBALD1	-1.06	bta-miR-1343-5p	ATG9A	-0.8
bta-miR-3141	SCRT1	-1.05	bta-miR-1343-5p	HMGA1	-0.8
bta-miR-2899	LENG8	-1.05	bta-miR-1246	ZNF23	-0.8
bta-miR-2888	AL359878.1	-1.03	bta-miR-2374	C6orf211	-0.79
bta-miR-2899	REPIN1	-1.03	bta-miR-2893	ZNF784	-0.79
bta-miR-1343-5p	RGMA	-1.03	bta-miR-2893	DBNDD2	-0.79
bta-miR-1584-5p	LCE3E	-1.02	bta-miR-2893	RP11-93B14.6	-0.79
bta-miR-2899	TBC1D14	-1.02	bta-miR-2899	SPI1	-0.79
bta-miR-2899	MTA1	-1.01	bta-miR-2899	PPIB	-0.79
bta-miR-2899	FAM222B	-1.01	bta-miR-2899	RECQL5	-0.79
bta-miR-2888	HSPB7	-1	bta-miR-2899	CNDP2	-0.79
bta-miR-2888	ERN1	-1	bta-miR-2899	C1orf35	-0.79
bta-miR-2888	STON2	-1	bta-miR-2899	C17orf103	-0.79
bta-miR-2888	SPTBN1	-1	bta-miR-2899	HEYL	-0.79
bta-miR-2888	LMNB2	-1	bta-miR-1343-5p	TBC1D13	-0.79
bta-miR-2899	MADD	-1	bta-miR-1343-5p	GRB7	-0.79
bta-miR-2899	TRABD	-1	bta-miR-2328-3p	hsa-mir-1199	-0.79
bta-miR-2899	SCRT1	-1	bta-miR-1584-5p	MTX2	-0.78
bta-miR-1343-5p	OSM	-1	bta-miR-1584-5p	PLEKHF1	-0.78
bta-miR-149-3p	FBXW8	-1	bta-miR-1584-5p	ACYP1	-0.78

bta-miR-2899	CORO7-PAM16	-0.98	bta-miR-2888	CTDSP1	-0.78
bta-miR-1584-5p	ZNF740	-0.97	bta-miR-2899	PDLIM2	-0.78
bta-miR-2899	MKNK2	-0.97	bta-miR-2899	HID1	-0.78
bta-miR-2899	RAB8A	-0.97	bta-miR-1343-5p	KLK14	-0.78
bta-miR-2899	MGLL	-0.97	bta-miR-1343-5p	UBE2QL1	-0.78
bta-miR-2899	NFAM1	-0.95	bta-miR-2887	AC093802.1	-0.78
bta-miR-1343-5p	LYPD1	-0.95	bta-miR-1584-5p	LY6E	-0.77
bta-miR-1343-5p	PKD1	-0.95	bta-miR-1584-5p	GEMIN4	-0.77
bta-miR-1343-5p	GPA33	-0.95	bta-miR-1584-5p	GPRIN1	-0.77
bta-miR-1343-5p	MS4A15	-0.95	bta-miR-2888	CENPB	-0.77
bta-miR-1584-5p	LCE3D	-0.94	bta-miR-2888	ZNF225	-0.77
bta-miR-2888	MUC5B	-0.94	bta-miR-2374	S100A11	-0.77
bta-miR-2328-3p	TMCC1	-0.94	bta-miR-2374	FAM107A	-0.77
bta-miR-3141	PRKAB1	-0.93	bta-miR-1343-5p	THTPA	-0.77
bta-miR-1584-5p	CD37	-0.93	bta-miR-1343-5p	PIRT	-0.77
bta-miR-1584-5p	GNAT1	-0.93	bta-miR-1343-5p	TNS4	-0.77
bta-miR-2899	TCP11	-0.93	bta-miR-1343-5p	HEPACAM	-0.77
bta-miR-2899	GRIN1	-0.93	bta-miR-1343-5p	STIM1	-0.77
bta-miR-2887	RPAIN	-0.93	bta-miR-2328-3p	KCTD5	-0.77
bta-miR-1584-5p	FAM222B	-0.92	bta-miR-3141	C17orf107	-0.76
bta-miR-2888	PCSK1N	-0.92	bta-miR-2888	INSL3	-0.76
bta-miR-2893	AC008948.1	-0.92	bta-miR-2888	IQSEC2	-0.76
bta-miR-2899	DLX1	-0.92	bta-miR-2899	IGLON5	-0.76
bta-miR-2899	ZCCHC24	-0.92	bta-miR-2899	ZC3H3	-0.76
bta-miR-1343-5p	COTL1	-0.92	bta-miR-1343-5p	RRP1	-0.76
bta-miR-2893	SLC7A8	-0.91	bta-miR-1343-5p	PACRG	-0.76
bta-miR-2899	FAM127A	-0.91	bta-miR-1343-5p	PSORS1C1	-0.76
bta-miR-1343-5p	FKBP1A	-0.91	bta-miR-1343-5p	GAREML	-0.76
bta-miR-1343-5p	HOXB5	-0.91	bta-miR-2328-3p	ZRSR1	-0.76
bta-miR-2887	BHLHA15	-0.91	bta-miR-1246	ORC6	-0.76
bta-miR-2887	SLC44A3	-0.91	bta-miR-1584-5p	NFIX	-0.75
bta-miR-3141	MAPK11	-0.9	bta-miR-2899	NRG2	-0.75
bta-miR-2888	SLC25A23	-0.9	bta-miR-2899	VWA1	-0.75
bta-miR-2893	BOD1L2	-0.9	bta-miR-2899	CCDC74A	-0.75
bta-miR-2374	VAMP2	-0.89	bta-miR-2899	LTBP4	-0.75
bta-miR-2899	RP11-20I23.1	-0.89	bta-miR-2899	FOSB	-0.75
bta-miR-3141	POU2F2	-0.88	bta-miR-1343-5p	NUPR1	-0.75
bta-miR-2888	PAX5	-0.88	bta-miR-1343-5p	SERPINE3	-0.75
bta-miR-2888	SYCE2	-0.88	bta-miR-1343-5p	AQP5	-0.75
bta-miR-2899	GRM4	-0.88	bta-miR-1343-5p	TSTA3	-0.75

bta-miR-2899	MS4A15	-0.88	bta-miR-1343-5p	GLIS1	-0.75
bta-miR-2899	TAGLN	-0.88	bta-miR-2887	TOR2A	-0.75
bta-miR-2899	C20orf194	-0.88	bta-miR-2887	GORASP1	-0.75
bta-miR-1246	FUT9	-0.88	bta-miR-1246	AL355390.1	-0.75
bta-miR-3141	FAM19A5	-0.87			

S. Table 5. Predicted mRNA targets of DEM common to 2-cell, 8-cell, blastocyst SM

miRNA	Predicted mRNAs	Cumulative Context Score	miRNAs	Predicted mRNAs	Cumulative Context Score
bta-miR-1777b	44080	-0.81	bta-miR-2900	MAPKAPK2	-1.4
bta-miR-1777b	44083	-1.39	bta-miR-1777b	MAPKAPK2	-0.96
bta-miR-1777a	ABCF2	-0.86	bta-miR-2885	MARK4	-0.83
bta-miR-1777b	AC007040.11	-0.75	bta-miR-1777b	MARVELD1	-1.15
bta-miR-2900	AC026202.1	-1	bta-miR-1777a	MASP2	-0.82
bta-miR-1777b	AC026202.1	-0.96	bta-miR-2305	MDGA1	-2.37
bta-miR-2305	AC068987.1	-1.07	bta-miR-1777b	MESDC1	-0.86
bta-miR-2305	AC093802.1	-0.89	bta-miR-1777b	METTL7B	-0.77
bta-miR-1777b	ACOX3	-1.08	bta-miR-2900	MEX3B	-0.81
bta-miR-1777a	ACTN4	-0.95	bta-miR-2305	MEX3C	-0.79
bta-miR-1777b	ACTR3B	-0.81	bta-miR-2305	MFGE8	-0.95
bta-miR-2900	ADAM11	-0.82	bta-miR-2900	MFGE8	-0.93
bta-miR-2305	ADAMTS10	-0.79	bta-miR-2305	MGAT5	-0.83
bta-miR-2900	ADAMTS13	-0.79	bta-miR-2900	MIDN	-1.42
bta-miR-1777b	ADD1	-0.75	bta-miR-1777b	MIDN	-0.78
bta-miR-2305	ADRM1	-0.83	bta-miR-2900	MIF	-0.95
bta-miR-2305	AF196779.12	-1.14	bta-miR-2900	MKNK2	-1.86
bta-miR-2305	AGAP1	-1.22	bta-miR-1777b	MKNK2	-0.85
bta-miR-1777a	AGFG2	-1.02	bta-miR-2305	MKNK2	-0.79
bta-miR-2885	AGFG2	-0.95	bta-miR-1777a	MLLT6	-1.17
bta-miR-2305	AGPAT3	-1.24	bta-miR-1777a	MMP15	-0.75
bta-miR-2900	AHDC1	-1.25	bta-miR-2305	MMP2	-1.03
bta-miR-2305	AIF1L	-0.85	bta-miR-2900	MS4A15	-1.56
bta-miR-2305	AK8	-0.78	bta-miR-1777b	MS4A15	-1.23
bta-miR-1777b	AKNA	-0.81	bta-miR-1777a	MSI1	-2.29
bta-miR-2900	AL117190.3	-0.85	bta-miR-2305	MSI1	-1.44
bta-miR-1777b	AL117190.3	-0.83	bta-miR-1777b	MSI1	-0.76
bta-miR-1777a	AL117190.3	-0.75	bta-miR-1777a	MTSS1L	-1.34
bta-miR-2885	AL450307.1	-0.82	bta-miR-2305	MTSS1L	-1.09

bta-miR-2885	AL590822.1	-0.75	bta-miR-2305	MUC5B	-2.19
bta-miR-2305	ANAPC15	-0.78	bta-miR-1777b	MUL1	-0.94
bta-miR-2900	ANK1	-1.03	bta-miR-2900	MUL1	-0.85
bta-miR-1777a	ANK1	-0.96	bta-miR-2900	MVB12B	-0.8
bta-miR-1777b	ANK1	-0.96	bta-miR-2900	MXD4	-1.17
bta-miR-2305	ANKRD13B	-0.86	bta-miR-2885	MZF1	-0.86
bta-miR-1777a	ANKRD52	-1.44	bta-miR-1777b	NAB2	-0.82
bta-miR-2305	ANKRD52	-0.81	bta-miR-1777a	NACC1	-0.97
bta-miR-2900	AP000350.10	-0.85	bta-miR-1777b	NANOS3	-0.75
bta-miR-2900	AP000350.4	-1.04	bta-miR-2900	NAPRT1	-0.77
bta-miR-2305	AP001816.1	-0.75	bta-miR-2900	NAT8L	-1.96
bta-miR-2900	ARC	-1.72	bta-miR-2305	NCF1	-0.92
bta-miR-1777b	ARC	-1.21	bta-miR-2305	NCKAP5L	-0.78
bta-miR-1777a	ARHGAP1	-0.83	bta-miR-1777a	NFAM1	-0.83
bta-miR-2900	ARHGAP17	-1.01	bta-miR-2305	NFIC	-1.67
bta-miR-1777a	ARHGAP17	-0.99	bta-miR-2305	NFIX	-0.75
bta-miR-1777b	ARHGAP17	-0.85	bta-miR-2305	NGB	-0.94
bta-miR-2900	ARHGAP23	-0.79	bta-miR-2305	NKAIN1	-1.13
bta-miR-2900	ARHGAP39	-0.93	bta-miR-2305	NKAIN4	-0.79
bta-miR-1777a	ATN1	-1.33	bta-miR-2885	NKX2-5	-0.8
bta-miR-1777b	ATN1	-1.23	bta-miR-2900	NODAL	-0.85
bta-miR-2900	ATN1	-1.07	bta-miR-2305	NOP2	-0.88
bta-miR-1777a	ATP1A3	-1.09	bta-miR-2900	NPTX2	-1.41
bta-miR-2900	ATP1A3	-0.78	bta-miR-2305	NPTXR	-0.76
bta-miR-1777b	ATP1A3	-0.76	bta-miR-2900	NRBP1	-0.83
bta-miR-1777a	ATP1B2	-0.8	bta-miR-1777b	NRGN	-1.1
bta-miR-2900	ATP6V0C	-1.46	bta-miR-1777a	NRGN	-1.06
bta-miR-1777b	ATP6V0C	-0.84	bta-miR-2900	NTNG2	-0.91
bta-miR-2900	AVPR2	-1.07	bta-miR-2305	NUMBL	-0.92
bta-miR-1777b	AVPR2	-0.97	bta-miR-1777b	NUP210	-0.78
bta-miR-2900	B3GAT1	-1.21	bta-miR-2305	NXF1	-1.13
bta-miR-2900	BANP	-0.91	bta-miR-2305	ONECUT3	-1.97
bta-miR-2305	BCL2L1	-2	bta-miR-1777a	ONECUT3	-1.27
bta-miR-2305	BEAN1	-1.52	bta-miR-1777a	OTP	-1.05
bta-miR-2305	BFSP2	-0.76	bta-miR-1777b	OTP	-1.02
bta-miR-2305	BGN	-0.8	bta-miR-2305	P2RX6	-0.96
bta-miR-1777a	BMP1	-1.29	bta-miR-2900	PACSIN1	-1.05
bta-miR-1777b	BOK	-0.91	bta-miR-2305	PACSIN1	-0.92
bta-miR-2305	BPIFA2	-0.75	bta-miR-2885	PACSIN2	-0.75
bta-miR-1777a	BRSK1	-0.84	bta-miR-2900	PAM16	-1.38

bta-miR-2900	BRSK2	-1.6	bta-miR-1777b	PAX2	-2.16
bta-miR-2900	BTBD9	-0.94	bta-miR-2900	PAX2	-2.15
bta-miR-1777b	C10orf55	-0.91	bta-miR-2305	PCDHGA1	-1.21
bta-miR-2305	C15orf52	-0.92	bta-miR-2305	PCDHGA10	-1.21
bta-miR-2305	C17orf103	-1.07	bta-miR-2305	PCDHGA11	-1.18
bta-miR-2900	C17orf107	-1.02	bta-miR-2305	PCDHGA12	-1.21
bta-miR-2305	C17orf50	-0.83	bta-miR-2305	PCDHGA2	-1.21
bta-miR-1777b	C17orf50	-0.77	bta-miR-2305	PCDHGA3	-1.21
bta-miR-1777b	C17orf74	-0.87	bta-miR-2305	PCDHGA4	-1.21
bta-miR-2305	C19orf43	-0.84	bta-miR-2305	PCDHGA5	-1.21
bta-miR-2900	C19orf73	-1.04	bta-miR-2305	PCDHGA6	-1.21
bta-miR-1777b	C1QL1	-0.88	bta-miR-2305	PCDHGA7	-1.21
bta-miR-2305	C1QL4	-0.75	bta-miR-2305	PCDHGA8	-1.21
bta-miR-2305	C20orf112	-0.77	bta-miR-2305	PCDHGA9	-1.21
bta-miR-2305	C21orf67	-1.19	bta-miR-2305	PCDHGB1	-1.22
bta-miR-2305	C22orf23	-0.75	bta-miR-2305	PCDHGB2	-1.21
bta-miR-2305	C22orf26	-0.83	bta-miR-2305	PCDHGB3	-1.21
bta-miR-2885	C3orf20	-0.79	bta-miR-2305	PCDHGB4	-1.22
bta-miR-2900	C3orf27	-0.76	bta-miR-2305	PCDHGB6	-1.21
bta-miR-2900	C6orf223	-0.92	bta-miR-2305	PCDHGB7	-1.21
bta-miR-2305	C7orf41	-0.76	bta-miR-2305	PCDHGC3	-1.21
bta-miR-1777a	C9orf62	-1.12	bta-miR-2305	PCDHGC4	-1.21
bta-miR-2885	CABP1	-0.83	bta-miR-2305	PCDHGC5	-1.65
bta-miR-2900	CABP7	-1.1	bta-miR-2305	PDE4A	-1.14
bta-miR-1777a	CACNG7	-1.4	bta-miR-1777a	PEX14	-0.78
bta-miR-2900	CACNG7	-0.98	bta-miR-2900	PEX6	-0.75
bta-miR-450b	CAMK2N1	-1.01	bta-miR-2305	PGAP3	-1.28
bta-miR-1777a	CAPN15	-0.8	bta-miR-2305	PHOX2B	-0.97
bta-miR-1777b	CAPN15	-0.75	bta-miR-2900	PIAS4	-0.78
bta-miR-2305	CASKIN2	-0.79	bta-miR-2900	PIRT	-0.98
bta-miR-2305	CBLN3	-0.81	bta-miR-1777a	PITX1	-0.87
bta-miR-2900	CBX6	-2.03	bta-miR-2305	PKNOX2	-1.02
bta-miR-1777b	CBX6	-1.18	bta-miR-2885	PLA2G1B	-1.08
bta-miR-2900	CBX7	-1.5	bta-miR-2305	PLA2G2F	-0.91
bta-miR-1777b	CBX7	-0.95	bta-miR-1777b	PLEKHO2	-0.78
bta-miR-2885	CCDC64	-0.8	bta-miR-2900	PLXNA1	-0.82
bta-miR-1777b	CCDC74A	-0.81	bta-miR-2900	PODXL2	-1.03
bta-miR-1777b	CCL3L1	-0.75	bta-miR-1777b	PODXL2	-0.82
bta-miR-2900	CD248	-0.79	bta-miR-2305	POLR1A	-0.98
bta-miR-2900	CD300LB	-0.85	bta-miR-2900	POLR2J2	-1.46

bta-miR-1777a	CDIP1	-0.92	bta-miR-2900	POLR2L	-0.81
bta-miR-2305	CDK5R1	-0.75	bta-miR-2900	POU2F2	-1.46
bta-miR-1777b	CDK5R2	-0.91	bta-miR-2900	PPARD	-1.02
bta-miR-2305	CDKN1A	-1.28	bta-miR-1777a	PPARD	-0.93
bta-miR-2305	CDR2L	-0.89	bta-miR-1777b	PPARD	-0.84
bta-miR-1777b	CDYL2	-0.92	bta-miR-1777b	PPFIA3	-0.9
bta-miR-2305	CEACAM19	-1.14	bta-miR-2900	PPP1R12C	-1
bta-miR-1777a	CECR1	-0.88	bta-miR-1777a	PPP1R3B	-0.77
bta-miR-2305	CELF5	-1.21	bta-miR-2305	PRAF2	-1.23
bta-miR-2900	CEP170B	-1.71	bta-miR-2900	PRKAB1	-0.8
bta-miR-2900	CERS1	-1.6	bta-miR-2900	PRKACA	-0.81
bta-miR-1777a	CERS1	-1.31	bta-miR-2900	PRKCG	-1.14
bta-miR-1777b	CERS1	-0.94	bta-miR-1777b	PRKCG	-0.81
bta-miR-2885	CERS1	-0.83	bta-miR-2305	PRR3	-0.75
bta-miR-2305	CIC	-0.84	bta-miR-2900	PRSS36	-0.84
bta-miR-1777a	CITED4	-1.27	bta-miR-2900	PSAPL1	-0.77
bta-miR-1777b	CKM	-1.05	bta-miR-2305	PSMD8	-0.77
bta-miR-2900	CKM	-0.89	bta-miR-2885	PTBP1	-0.75
bta-miR-2305	CLDN2	-0.92	bta-miR-2900	PTK2B	-1.14
bta-miR-2305	CLIP3	-1.27	bta-miR-1777b	PTK2B	-0.98
bta-miR-1777b	CLPP	-0.82	bta-miR-2305	PTPN7	-1.02
bta-miR-1777b	CNDP2	-0.8	bta-miR-2900	PTPRS	-0.97
bta-miR-2900	CNFN	-1	bta-miR-1777a	R3HDM4	-0.81
bta-miR-1777a	CNFN	-0.98	bta-miR-1777b	RAB11B	-1.11
bta-miR-1777b	CNFN	-0.95	bta-miR-2900	RAB11B	-0.87
bta-miR-2305	CNIH2	-1.01	bta-miR-2305	RAB3A	-1.66
bta-miR-2900	CNOT3	-1.29	bta-miR-2900	RAB8A	-1.7
bta-miR-2305	CNPY3	-0.84	bta-miR-2900	RAD51L3-RFFL	-0.78
bta-miR-2900	CNTFR	-0.82	bta-miR-2900	RADIL	-1.41
bta-miR-2305	COMMD7	-0.86	bta-miR-2305	RAPGEFL1	-0.89
bta-miR-2900	COPS7A	-0.8	bta-miR-2900	RASL10B	-1.39
bta-miR-2305	COPS7A	-0.76	bta-miR-2305	RASL10B	-0.82
bta-miR-2305	COPZ1	-1.18	bta-miR-1777b	RASL10B	-0.77
bta-miR-2900	CORO7-PAM16	-1.13	bta-miR-2305	RAX	-1.21
bta-miR-2900	COTL1	-0.78	bta-miR-2885	RAX	-0.91
bta-miR-1777b	COX6B2	-1.61	bta-miR-2305	RBFOX3	-1.08
bta-miR-2900	CRIP2	-1.77	bta-miR-2900	RCVRN	-1.13
bta-miR-2305	CRMP1	-0.75	bta-miR-1777b	REM2	-0.8
bta-miR-1777a	CRTC1	-0.81	bta-miR-1777a	RGMA	-2.44
bta-miR-2305	CSDC2	-1.23	bta-miR-2885	RGMA	-1.23

bta-miR-1777a	CTIF	-0.95	bta-miR-2305	RGMA	-0.96
bta-miR-1777b	CTIF	-0.9	bta-miR-2305	RHO	-0.75
bta-miR-2305	CTRL	-0.81	bta-miR-2900	RHOD	-0.78
bta-miR-1777a	CTXN1	-0.97	bta-miR-2305	RHOG	-0.87
bta-miR-2305	CX3CL1	-1.33	bta-miR-2305	RNF144A	-0.76
bta-miR-1777b	CYB5R3	-1.16	bta-miR-2305	RNF185	-0.76
bta-miR-2305	CYP2R1	-0.88	bta-miR-2900	RNF222	-0.79
bta-miR-1777a	CYP2S1	-1.12	bta-miR-2305	RNF44	-1.56
bta-miR-2305	CYP3A4	-0.82	bta-miR-2305	RNPS1	-1.43
bta-miR-2305	CYP46A1	-1.49	bta-miR-2305	ROGDI	-0.91
bta-miR-1777b	CYP4F22	-0.83	bta-miR-1777b	RP11-195F19.5	-1.04
bta-miR-2305	DAB2IP	-1.01	bta-miR-2900	RP11-195F19.5	-0.75
bta-miR-2900	DAB2IP	-0.81	bta-miR-2900	RP11-20I23.1	-1.12
bta-miR-2305	DAGLA	-1.21	bta-miR-2305	RP11-247C2.2	-0.8
bta-miR-2900	DDA1	-1.07	bta-miR-2900	RP11-527L4.2	-1.41
bta-miR-1777a	DDR1	-0.76	bta-miR-2305	RP11-94B19.4	-1.01
bta-miR-1777b	DDX39B	-0.85	bta-miR-2305	RPH3A	-1.13
bta-miR-2305	DERL3	-1.02	bta-miR-1777a	RPS6KA2	-1.37
bta-miR-1777b	DET1	-0.76	bta-miR-2900	RPS6KA2	-0.84
bta-miR-1777a	DKFZP761J1410	-0.88	bta-miR-1777b	RPS6KA2	-0.8
bta-miR-2885	DKFZP779J2370	-0.78	bta-miR-2305	RPUSD1	-1.14
bta-miR-1777b	DLGAP3	-0.79	bta-miR-2305	RUNX3	-0.94
bta-miR-2900	DLGAP3	-0.76	bta-miR-2305	SAMD4B	-0.93
bta-miR-1777a	DNAH17-AS1	-0.95	bta-miR-1777b	SBK1	-1.1
bta-miR-1777b	DNAJC4	-0.96	bta-miR-2900	SBK1	-0.82
bta-miR-2305	DPM2	-0.8	bta-miR-2900	SCN1B	-0.8
bta-miR-1777a	DUSP16	-0.87	bta-miR-2900	SCRT1	-2.53
bta-miR-1777b	DUSP22	-0.78	bta-miR-1777b	SCRT1	-1.52
bta-miR-2900	ECE1	-0.77	bta-miR-1777a	SDK2	-0.9
bta-miR-1777b	EEF1A2	-0.86	bta-miR-1777b	SEC61A1	-0.79
bta-miR-1777a	EGLN3	-0.83	bta-miR-2900	SEPT9	-1.02
bta-miR-1777b	EGLN3	-0.8	bta-miR-2305	SETD1B	-2.24
bta-miR-2900	EHD2	-1.29	bta-miR-2900	SGSM1	-1.02
bta-miR-1777b	EHD2	-1.02	bta-miR-1777a	SHOX	-1.01
bta-miR-2305	EIF2AK1	-0.76	bta-miR-2305	SIPA1L3	-1.22
bta-miR-2305	ELAVL3	-0.97	bta-miR-1777b	SIPA1L3	-0.8
bta-miR-2305	ELK1	-0.88	bta-miR-2305	SIT1	-0.8
bta-miR-2900	ELN	-1.73	bta-miR-1777b	SIVA1	-0.96
bta-miR-2305	ENG	-0.78	bta-miR-1777b	SLC12A9	-0.77
bta-miR-2900	EPHA8	-0.92	bta-miR-2305	SLC17A7	-0.88

bta-miR-1777a	EPHB2	-0.79	bta-miR-2305	SLC25A23	-1.17
bta-miR-1777a	EPS8L2	-1.43	bta-miR-2885	SLC25A28	-1.02
bta-miR-2305	ERF	-0.78	bta-miR-2900	SLC29A4	-0.85
bta-miR-1777b	EXOSC5	-0.94	bta-miR-1777a	SLC41A1	-0.8
bta-miR-2305	FAM131B	-0.77	bta-miR-2305	SLC45A3	-0.96
bta-miR-1777a	FAM132A	-0.98	bta-miR-2305	SLC48A1	-0.99
bta-miR-2305	FAM155B	-0.92	bta-miR-2305	SLC6A1	-1.52
bta-miR-1777b	FAM19A5	-1.07	bta-miR-2305	SLC6A17	-0.86
bta-miR-2900	FAM19A5	-0.97	bta-miR-1777b	SLC6A8	-1
bta-miR-2885	FAM219A	-0.75	bta-miR-1777b	SLC7A1	-0.83
bta-miR-2305	FAM222B	-2.06	bta-miR-2305	SMARCC2	-0.8
bta-miR-2305	FAM43B	-1.12	bta-miR-2305	SMR3B	-0.79
bta-miR-1777b	FAM53B	-0.75	bta-miR-2900	SNCB	-0.75
bta-miR-2305	FAM57B	-0.92	bta-miR-1777b	SOD3	-1
bta-miR-2885	FAM73B	-0.86	bta-miR-1777a	SOD3	-0.96
bta-miR-2305	FAM83F	-0.82	bta-miR-2305	SORCS2	-2
bta-miR-2900	FBXL16	-1.95	bta-miR-1777a	SOST	-1.2
bta-miR-2900	FBXL18	-0.86	bta-miR-1777b	SOST	-0.9
bta-miR-2305	FBXO46	-1.65	bta-miR-1777a	SOX12	-1.11
bta-miR-2305	FEV	-0.9	bta-miR-2305	SOX15	-0.81
bta-miR-2305	FGF4	-0.76	bta-miR-1777b	SOX3	-1.46
bta-miR-2305	FIBCD1	-1.49	bta-miR-2305	SPEG	-0.87
bta-miR-2305	FKBP8	-1.06	bta-miR-1777b	SPI1	-1.05
bta-miR-1777b	FLOT2	-0.85	bta-miR-2305	SPRED2	-0.76
bta-miR-2900	FOXJ2	-0.81	bta-miR-2900	SPRN	-0.9
bta-miR-2305	FOXP4	-0.79	bta-miR-1777b	SPRN	-0.82
bta-miR-1777a	FRMPD3	-0.95	bta-miR-1777a	SPRN	-0.77
bta-miR-2900	FUOM	-1.21	bta-miR-2305	SPRY4	-1.35
bta-miR-2305	FXYD1	-0.8	bta-miR-2885	SPSB4	-0.81
bta-miR-1777b	FXYD6	-1.28	bta-miR-2305	SPTB	-0.79
bta-miR-2900	FXYD6	-1.14	bta-miR-2900	SRRM4	-0.77
bta-miR-2305	G6PD	-1.32	bta-miR-2305	SSBP3-AS1	-0.99
bta-miR-1777b	GAL	-0.78	bta-miR-2305	ST3GAL3	-0.81
bta-miR-2900	GAS8	-1.09	bta-miR-2305	STX1A	-0.99
bta-miR-1777b	GAS8	-0.99	bta-miR-2900	STX1A	-0.76
bta-miR-1777b	GATA4	-1.2	bta-miR-2900	SUCLG1	-0.76
bta-miR-2900	GATSL2	-0.8	bta-miR-2305	SUV420H2	-1.3
bta-miR-1777b	GNA12	-0.91	bta-miR-2305	SYNGAP1	-1.04
bta-miR-1777a	GOLGA2	-0.84	bta-miR-1777a	SYNGR1	-0.94
bta-miR-2305	GORASP1	-1.48	bta-miR-1777a	TBC1D22B	-1

bta-miR-1777b	GORASP1	-1.15	bta-miR-2305	TBKBP1	-0.82
bta-miR-1777b	GPR144	-0.91	bta-miR-1777a	TBX10	-0.88
bta-miR-2305	GPS1	-1.15	bta-miR-1777a	TBX5	-0.97
bta-miR-2305	GPSM1	-0.87	bta-miR-2900	TBX5	-0.81
bta-miR-1777b	GSE1	-0.75	bta-miR-2305	TBX6	-0.85
bta-miR-2900	GTF3C5	-0.92	bta-miR-2900	TCF7L2	-1.02
bta-miR-2305	HCN2	-0.97	bta-miR-2305	TEAD2	-0.93
bta-miR-1777b	HCN2	-0.84	bta-miR-2900	TFF3	-0.88
bta-miR-1777a	HDGF	-0.8	bta-miR-2900	TGM2	-0.96
bta-miR-1777a	HIF3A	-0.96	bta-miR-2305	THRA	-1.26
bta-miR-2305	HIPK2	-0.78	bta-miR-2900	THRA	-0.78
bta-miR-1777a	HIRIP3	-1.21	bta-miR-2305	THTPA	-0.75
bta-miR-2305	HMGA1	-1.04	bta-miR-2305	TIMP2	-0.77
bta-miR-2305	HNF4A	-0.87	bta-miR-1777b	TLX3	-1.41
bta-miR-2900	HOXA3	-1.66	bta-miR-2900	TLX3	-0.79
bta-miR-2305	HOXA7	-0.85	bta-miR-2305	TMEM105	-0.75
bta-miR-2305	HOXB1	-1.44	bta-miR-2900	TMEM127	-1.05
bta-miR-2900	HOXB8	-0.77	bta-miR-1777b	TMEM127	-0.82
bta-miR-2305	HOXC12	-1.32	bta-miR-2305	TMEM201	-1.21
bta-miR-1777a	HOXC6	-1.35	bta-miR-2305	TMEM213	-0.75
bta-miR-2900	HOXC6	-1.03	bta-miR-2305	TMEM229B	-0.78
bta-miR-2305	HPCA	-0.75	bta-miR-2305	TMEM249	-0.75
bta-miR-2900	HPD	-2.44	bta-miR-2305	TMEM257	-0.76
bta-miR-2305	HRK	-0.88	bta-miR-1777a	TMEM63C	-1.21
bta-miR-1777a	HS3ST3B1	-0.93	bta-miR-1777b	TMEM63C	-0.76
bta-miR-2900	HSPB6	-1.43	bta-miR-2305	TNFRSF12A	-0.77
bta-miR-2305	HSPB7	-1.85	bta-miR-2305	TNNT2	-1.08
bta-miR-1777a	HSPG2	-0.98	bta-miR-1777a	TNRC18	-2.02
bta-miR-2305	HTR5A-AS1	-0.96	bta-miR-2305	TNS1	-0.75
bta-miR-1777a	IFITM2	-0.88	bta-miR-1777a	TOM1L2	-0.96
bta-miR-2305	IGDCC3	-0.88	bta-miR-2305	TP53I11	-0.76
bta-miR-1777a	IGF1R	-0.81	bta-miR-2305	TPBGL	-0.97
bta-miR-2305	IGF2	-1.2	bta-miR-2900	TRABD	-1.69
bta-miR-2305	IGFBP2	-0.9	bta-miR-1777b	TRABD	-1.1
bta-miR-2305	IKZF4	-1	bta-miR-1777a	TRABD	-0.82
bta-miR-2305	IQSEC2	-1.48	bta-miR-2900	TRAF3	-0.82
bta-miR-1777b	IQSEC3	-1	bta-miR-1777b	TRAF3	-0.77
bta-miR-2900	IQSEC3	-0.95	bta-miR-2900	TRMT61A	-0.77
bta-miR-1777a	IQSEC3	-0.82	bta-miR-2900	TRPV4	-1.08
bta-miR-2900	ISLR2	-0.77	bta-miR-1777b	TRPV4	-0.88

bta-miR-2305	KCNAB2	-1.2	bta-miR-1777b	TTYH3	-2.26
bta-miR-2305	KCNC1	-0.81	bta-miR-2900	TTYH3	-2.17
bta-miR-2305	KCNC3	-1.6	bta-miR-2305	TUBB3	-0.96
bta-miR-1777b	KCNIP3	-0.87	bta-miR-2305	TUBB4A	-0.87
bta-miR-2305	KCNK12	-0.79	bta-miR-2305	TUSC2	-1.17
bta-miR-2305	KCNK3	-1.04	bta-miR-2305	TUSC5	-0.76
bta-miR-2305	KCTD2	-1.08	bta-miR-1777b	UAP1L1	-1.33
bta-miR-1777b	KHSRP	-1.12	bta-miR-1777a	UAP1L1	-0.91
bta-miR-2900	KHSRP	-0.81	bta-miR-2900	UAP1L1	-0.84
bta-miR-2900	KIAA1671	-1.2	bta-miR-1777a	UBALD1	-0.93
bta-miR-1777a	KIF21B	-1.88	bta-miR-2305	UBALD1	-0.86
bta-miR-1777b	KIF21B	-1.84	bta-miR-2885	UBALD1	-0.76
bta-miR-2900	KIF21B	-1.71	bta-miR-2305	URM1	-1.06
bta-miR-2305	KIF21B	-0.88	bta-miR-2305	USB1	-1.34
bta-miR-1777b	KIFC2	-0.89	bta-miR-2305	VAMP1	-0.88
bta-miR-2900	KLC2	-1.41	bta-miR-2305	VAMP2	-1.66
bta-miR-2900	KLHL22	-0.94	bta-miR-2885	VAMP8	-0.81
bta-miR-2305	KMT2B	-0.9	bta-miR-2900	VGF	-1.17
bta-miR-2305	KMT2D	-2.79	bta-miR-1777a	VGF	-0.92
bta-miR-1777b	KRTAP4-4	-1.54	bta-miR-2305	VPS9D1	-1.11
bta-miR-1777b	KSR1	-0.87	bta-miR-2305	VSX2	-0.77
bta-miR-2305	KSR2	-1.26	bta-miR-2305	WDR45	-1.14
bta-miR-2305	LARP1	-1.14	bta-miR-2900	WDTC1	-0.8
bta-miR-1777a	LARS2	-0.77	bta-miR-2885	WNK2	-1.13
bta-miR-2900	LCN8	-0.78	bta-miR-1777a	WNT7B	-0.79
bta-miR-2305	LDB3	-0.79	bta-miR-2305	XKR7	-1.84
bta-miR-2305	LDHD	-0.84	bta-miR-2900	XYLT1	-1
bta-miR-1777a	LEMD2	-0.79	bta-miR-2885	YIF1B	-0.95
bta-miR-1777b	LEMD2	-0.76	bta-miR-1777b	YWHAH	-1.4
bta-miR-2885	LENG8	-0.85	bta-miR-2900	YWHAH	-0.82
bta-miR-2305	LIF	-1.19	bta-miR-2305	ZBTB7A	-2.18
bta-miR-2305	LIN37	-0.94	bta-miR-1777a	ZC3H4	-0.89
bta-miR-2900	LMOD1	-0.83	bta-miR-2305	ZC3H7B	-1.66
bta-miR-2305	LMTK3	-1.31	bta-miR-1777b	ZC3H7B	-0.92
bta-miR-2305	LMX1B	-1.74	bta-miR-2305	ZCCHC24	-1
bta-miR-2305	LRCH4	-1.11	bta-miR-2900	ZCCHC24	-0.94
bta-miR-2305	LRRC61	-1.35	bta-miR-2900	ZDHHC1	-0.89
bta-miR-1777a	LSP1	-0.84	bta-miR-1777a	ZDHHC8	-0.97
bta-miR-2900	LTBP4	-1.15	bta-miR-2305	ZFHX2	-0.85
bta-miR-2885	LTBP4	-0.83	bta-miR-2305	ZFP36L1	-1.46

bta-miR-1777b	LTBP4	-0.83	bta-miR-2305	ZNF385A	-1.47
bta-miR-2305	LY6K	-0.84	bta-miR-1777a	ZNF385A	-1.01
bta-miR-2305	MAF	-0.9	bta-miR-2900	ZNF385A	-0.99
bta-miR-2900	MAFK	-1.42	bta-miR-1777b	ZNF385A	-0.95
bta-miR-1777b	MAP2K7	-0.77	bta-miR-2305	ZNF648	-0.75
bta-miR-2900	MAP4	-0.75	bta-miR-2900	ZNF783	-0.82
bta-miR-2900	MAPK1	-1.03	bta-miR-2305	ZSWIM4	-1
bta-miR-2305	MAPK12	-0.97			
